# Programmable Information Processing With Reconfigurable Self‐Contact Variable Stiffness Mechanical Metamaterial

**DOI:** 10.1002/advs.77680

**Published:** 2026-09-08

**Authors:** Huadong Qin, Wenyu Zhu, Xin Wang, Cheng Shen, Li Cheng, Tian Jian Lu, Terence Xiaoteng Liu, Han Meng

**Affiliations:** ^1^ State Key Laboratory of Mechanics and Control for Aerospace Structures Nanjing University of Aeronautics and Astronautics Nanjing P. R. China; ^2^ MIIT Key Laboratory of Multifunctional Lightweight Materials and Structures Nanjing University of Aeronautics and Astronautics Nanjing P. R. China; ^3^ China Academy of Aerospace System and Innovation Beijing P. R. China; ^4^ Department of Mechanical Engineering The Hong Kong Polytechnic University Hong Kong P. R. China; ^5^ School of Engineering Physics and Mathematics Northumbria University Newcastle upon Tyne UK

**Keywords:** information processing, information storage and encryption, reconfiguration, self‐contact, tension‐compression asymmetry

## Abstract

Confronting the limitations of conventional electronics, such as electromagnetic interference and poor extreme environment reliability, mechanical metamaterials offer a promising pathway for robust physical information processing. A core challenge remains achieving dynamic, reversible stiffness programming. Here, we propose a reconfigurable self‐contact variable stiffness (SVS) metamaterial based on shape memory polymers (SMPs). The design features two unit cells with complementary asymmetry: one soft in tension/stiff in compression, and the other stiff in tension/soft in compression. By leveraging the shape memory effect, we precisely program the gap between internal contact blocks of the unit cell, actively controlling stiffness jumps and switching asymmetry modes. We demonstrate the platform's potential in two dimensions. First, an in‐plane array achieves information storage (465.9 KB/m^2^) and encryption. Crucially, it retains data integrity under strong electromagnetic fields, outperforming traditional electronics. Second, combining units into a four‐cell structure allows switchable asymmetric stiffness to convert symmetric vibrations into directional responses, realizing programmable matter transport (∼30 mm/s). This work provides a viable, robust mechanical solution for information processing in extreme environments where electronic systems are prone to failure.

## Graphical abstract

Reconfigurable self‐contact variable stiffness metamaterials based on shape memory polymers enable programmable stiffness switching. Static arrays store and encrypt information with immunity to electromagnetic interference, while asymmetric stiffness converts symmetric vibration into directional matter transport. This robust mechanical platform offers a viable information processing solution for extreme environments where electronic systems fail.

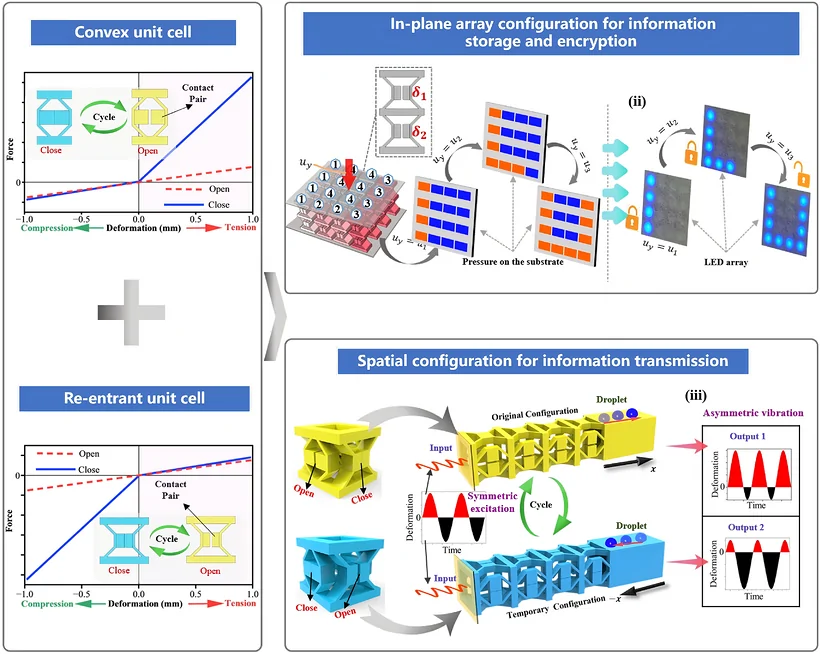

## 1 Introduction

Advances in information science rely on the continuous exploration of technologies for high‐efficiency, secure storage and rapid, precise transmission. Currently, mainstream information processing technologies are predominantly based on electronic principles. Despite their tremendous success in speed and integration, they are facing increasingly severe challenges in energy consumption, electromagnetic interference, and reliability in extreme environments. Consequently, researchers are actively pursuing novel information paradigms that differ from conventional electronic frameworks, such as information processing systems based on photons, ions, or mechanical media. Research on constructing physical information platforms using mechanical metamaterials holds unique value owing to their high robustness, interference immunity, and ability to interact directly with the physical world. Mechanical metamaterials can not only bear loads but also encode and transmit information, holding great promise to become a critical bridge connecting the digital and physical realms [1, 2, 3, 4, 5, 6, 7, 8].

However, transforming mechanical metamaterials from functional, static load‐bearing components into intelligent platforms capable of processing and manipulating information hinges critically on overcoming a core bottleneck: achieving dynamic and reversible programming of their mechanical properties, especially stiffness [9, 10]. As one of the most fundamental mechanical attributes of a structure, stiffness directly determines its load‐bearing capacity, deformation, and dynamic response [11, 12, 13, 14]. Traditional mechanical metamaterials exhibit fixed properties after fabrication, making them difficult to adapt to changing environments [15, 16]. To achieve stiffness modulation, researchers have explored various strategies, such as adjusting structural pre‐deformation [17, 18, 19, 20] and internal pressure [21, 22, 23], assembling discrete mechanical systems (e.g., gears) [24], and triggering configuration jumps in multi‐stable structures [15]. Recently, self‐contact variable stiffness (SVS) metamaterials have been proposed [25, 26, 27, 28]. Through ingenious configuration design, specific internal components of these metamaterials can come into contact with each other under load. Once the contact is established, it significantly alters the internal load‐transfer path, thereby causing a step‐change in the overall structural stiffness [29, 30]. As a purely geometric mechanical phenomenon, self‐contact offers distinct advantages over other stiffness tuning methods, including simple control, rapid response, and high robustness.

Furthermore, the self‐contact mechanism naturally endows the mechanical metamaterial with a unique and highly valuable mechanical property: tension‐compression stiffness asymmetry. This manifests as the structure's tensile stiffness being significantly greater than its compressive stiffness (i.e., “stiff in tension, soft in compression”) or, conversely, its compressive stiffness being significantly greater than its tensile stiffness (i.e., “soft in tension, stiff in compression”). This asymmetry originates from the asymmetric triggering of the self‐contact behavior under tensile vs. compressive loads. For instance, when internal components contact each other under compression, they squeeze against one another, thereby increasing compressive stiffness, whereas under tension, the components separate, resulting in lower tensile stiffness, thus exhibiting the “soft in tension, stiff in compression” behavior. The opposite holds true. Stiffness asymmetry breaks the traditional paradigm where a material exhibits the same response under oppositely directed loads. In fact, asymmetric stiffness materials also exist in nature [31]; for example, myocardial tissue exhibits different stiffness when sheared to left vs. right [32]. Stiffness asymmetry constitutes the fundamental physical basis for breaking system reciprocity [33], directly giving rise to distinctive properties such as non‐reciprocal force‐displacement responses (i.e., static non‐reciprocity) and non‐reciprocal wave Poynting effects [34]. Recently, as a research hotspot, scholars have successively proposed various asymmetric stiffness metamaterials. For example, fishbone‐shaped metamaterials exhibit tension‐compression asymmetry, enabling unidirectional displacement amplification in static systems [35]; nanocomposites with embedded fillers display asymmetric stiffness under shear, enabling the conversion of symmetric vibrations into asymmetric ones to achieve functions like energy harvesting and droplet manipulation [36]; and kirigami‐inspired non‐reciprocal metamaterials achieve multiple static non‐reciprocal modes (including shear and tension‐compression asymmetry) within a single microstructural topology by introducing controllable cuts and contact nonlinearity, thereby realizing functions like mechanical diodes and rectifiers [30].

Despite these advances, existing SVS metamaterials are fundamentally passive; their stiffness and asymmetry are preset during fabrication and cannot be reprogrammed afterward [30]. This “function‐fixed” nature severely limits their utility in intelligent systems. For instance, a fixed‐geometry array can store information but cannot update it, while a fixed‐asymmetry structure can only drive motion in one direction. Therefore, the immediate priority is to transition from the “passive and fixed” self‐contact mechanism to an “active and reconfigurable” one.

To address this challenge, we propose the deep integration of smart materials with the SVS mechanism to create truly reconfigurable metamaterials. We select shape memory polymers (SMPs) [37] for their large recoverable strain, lightweight nature, and multi‐stimuli responsiveness [38, 39, 40, 41, 42, 43, 44, 45, 46]. By fabricating convex and re‐entrant polygonal unit cells from SMPs, we leverage the shape memory effect to actively program the core parameter—the distance between self‐contact blocks. This innovation shifts control from passive response to active programming. The power of this approach lies in its direct alignment with two core requirements of information applications: First, for static information storage and encryption, our system provides addressable “0” and “1” states through discrete stiffness jumps. Programming the distances between self‐contact blocks, the critical load thresholds for these state changes, turning load value into a physical encryption key. The reconfigurability of the SMP allows for information erasure and rewriting, creating an ideal, programmable physical storage medium. Second, for dynamic information transmission, the core requirements are the generation of directional driving forces and the ability to exercise programmable control over this direction. It is noteworthy that in the field of information transmission, a powerful and robust strategy is to use matter itself as the information carrier, encoding and transmitting information by precisely controlling its position, movement path, or sequence. The tensile‐compressive stiffness asymmetry, a natural byproduct of the self‐contact mechanism in this study, is converted into asymmetry in response under vibrational excitation, thereby breaking the vibration symmetry and generating a net driving force. More importantly, reconfigurability allows the unit's asymmetric mode (e.g., “stiff in tension, soft in compression”) to be dynamically switched, thus enabling precise programmatic control over the dominant vibration direction, which is also the direction of matter transport.

This paper is structured as follows: Section 2 outlines the mechanical metamaterial's mechanism and its potential applications. Section 3 details the theoretical analysis and experimental validation of the mechanism. Sections 4 and 5 present the implementation and experimental verification of the metamaterial for static information storage/encryption and dynamic information transmission, respectively.

## 2 Structural Principle and Functionality

**FIGURE 1:**
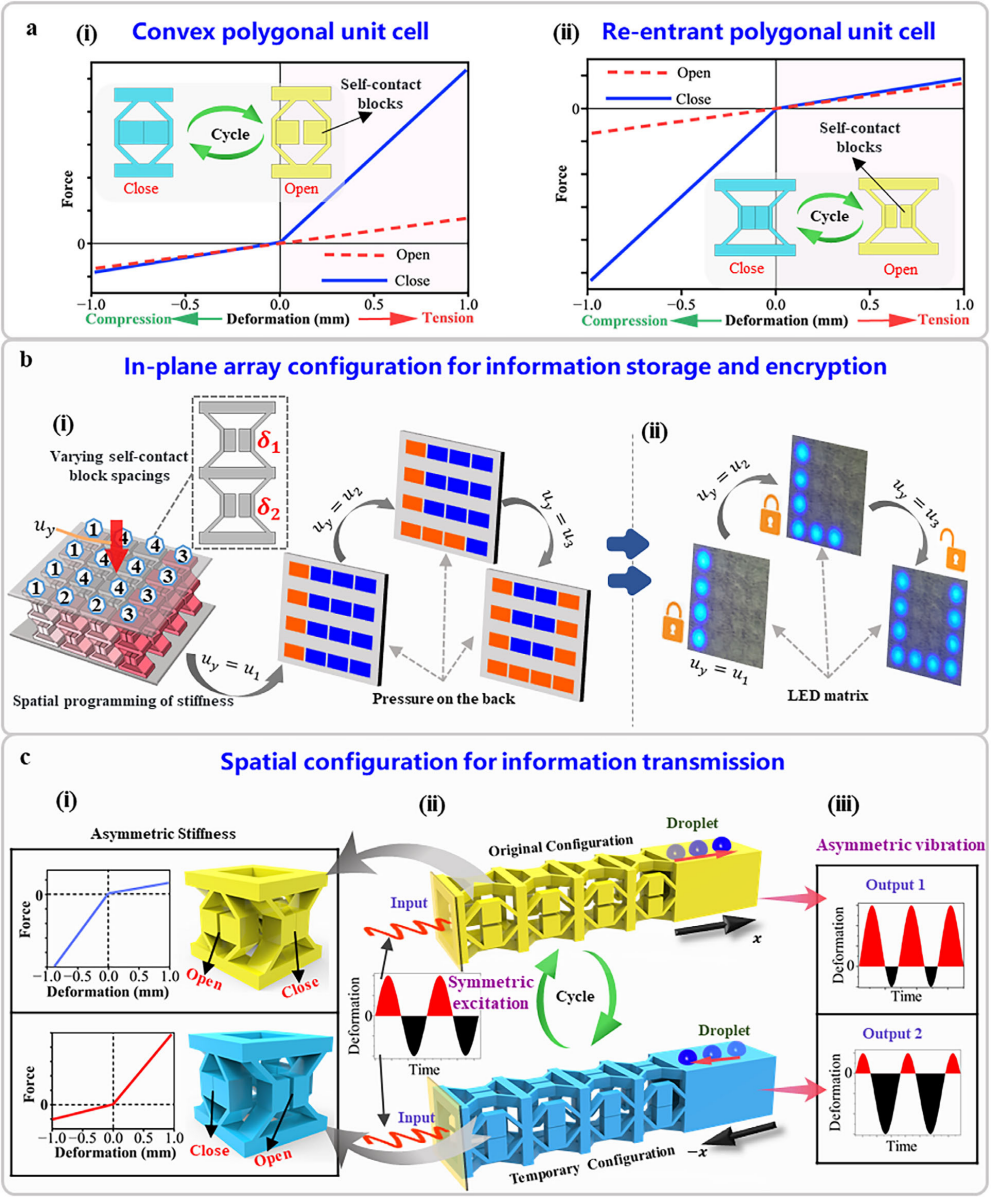
Design, performance, and functional applications of the reconfigurable SVS metamaterial. (a) Two basic units: (i) the convex polygonal unit cell with positive Poisson's ratio, exhibiting “stiff in tension, soft in compression” asymmetric stiffness when contact blocks are closed (blue solid line) and low stiffness in tension/compression when contact blocks are separated (red dashed line), (ii) the re‐entrant polygonal unit cell with negative Poisson's ratio, exhibiting “soft in tension, stiff in compression” asymmetric stiffness when self‐contact blocks are closed (blue solid line) and low stiffness when they are separated (red dashed line). (b) In‐plane array configuration for information storage and encryption: (i) spatial regulation of stiffness distribution and temporal control of abrupt stiffness changes via programming preset gaps (*δ*_1_, *δ*_2_) of individual units in periodic arrays. (ii) externally connecting an LED array to the metamaterial enables visualization of stored information. (c) Spatial configuration for information transmission: (i) a four‐cell metamaterial that integrates convex and re‐entrant polygonal unit cells, enabling active switching of asymmetric stiffness modes (switching between “stiff in tension and soft in compression” and “soft in tension and stiff in compression”) through geometric reconfiguration, (ii) metamaterial formed by axial series connection of the four‐cell metamaterial, (iii) the asymmetric stiffness of the metamaterial converts symmetric harmonic excitation into asymmetric vibration of the output platform.

The proposed reconfigurable SVS metamaterial comprises two differently shaped units: convex polygonal unit cell and re‐entrant polygonal unit cell (Figure 1a). Each unit is equipped with a pair of self‐contact blocks at its center. When subjected to out‐of‐plane loads, both units generate lateral displacement at their midsections. For the convex polygonal unit cell (Figure 1a(i)), under out‐of‐plane tensile loading, the self‐contact blocks move inward (i.e., lateral contraction under tension); conversely, the self‐contact blocks move outward under compression. For the re‐entrant polygonal unit cell (Figure 1a(ii)), under out‐of‐plane tensile loading, the self‐contact blocks move outward (i.e., lateral expansion under tension); conversely, the self‐contact blocks move inward under compression. For the convex polygonal unit cell with zero self‐contact block spacing (Figure 1a(i), “Close”), tensile loading causes the self‐contact blocks to contact and press against each other, significantly enhancing internal structural constraints and increasing tensile stiffness. Under compressive loading, however, the self‐contact blocks continuously separate with longitudinal deformation, resulting in no internal constraints and low compressive stiffness. This gives the “closed” convex polygonal unit cell a “stiff in tension, soft in compression” characteristic (Figure 1a(i), blue solid line). When the self‐contact block spacing is non‐zero (Figure 1a(i), “Open”), the unit exhibits identical tensile and compressive stiffness before contact occurs (Figure 1a(i), red dashed line). Similarly, the re‐entrant polygonal unit cell with zero self‐contact block spacing exhibits “soft in tension, stiff in compression” behavior (Figure 1a(ii), blue solid line), while non‐zero spacing results in symmetric tensile and compressive stiffness (Figure 1a(ii), red dashed line).

Through rational combination, the aforementioned SVS units can be spatially arranged in different configurations to form metamaterials with specific functions. As shown in Figure 1b, re‐entrant polygonal unit cells with varying self‐contact block spacings are arranged in‐plane to create metamaterials capable of information storage and encryption. Due to differing self‐contact block distances, units with smaller spacing contact first during deformation, causing localized stiffness increases that result in varying backside pressures at different metamaterial positions under compression. By presetting self‐contact block spacings in the unit array according to stored information, data storage is achieved. Additionally, pressure sensors, LEDs, and controllers are integrated on the metamaterial's backside; when backside pressure reaches specific thresholds, corresponding LEDs illuminate, enabling visual readout of stored information. Furthermore, since self‐contact block spacings of individual pixel units are preset to different values, varying compressive loads trigger different numbers and sequences of contacting units, resulting in distinct information patterns. Only by applying a specific compressive force as the “password” can correct information be read, enabling encrypted storage. Moreover, as the metamaterial is fabricated using shape memory materials, self‐contact block spacings can be reconfigured after information readout, allowing information erasure and rewriting.

In another application, the two kinds of unit cells (i.e., the convex and re‐entrant polygonal unit cells) are spatially combined to form four‐cell tension‐compression asymmetric metamaterials for directional transport of droplets or other substances (Figure 1c), providing a physical platform for information transmission as mentioned above. Owing to tension‐compression stiffness asymmetry, harmonic vibration excitation induces larger amplitudes in the lower‐stiffness direction and smaller amplitudes in the higher‐stiffness direction. Under combined inertial and asymmetric vibration effects, substances such as droplets attached to the metamaterial surface move unidirectionally, enabling directional transmission of information carriers. Furthermore, leveraging the shape memory effect of the constituent materials, the self‐contact block spacing of the unit cells can be switched between “closed” and “open” states, reversing the stiffness asymmetry (from “soft in tension, stiff in compression” to “stiff in tension, soft in compression” or vice versa). This allows programmable control of the asymmetric vibration direction (i.e., amplitude‐dominant direction), precisely regulating transport direction of substances, and laying the foundation for information transmitting and manipulation.

## 3 Mechanism and Verification of Reconfigurable SVS Metamaterials

**FIGURE 2:**
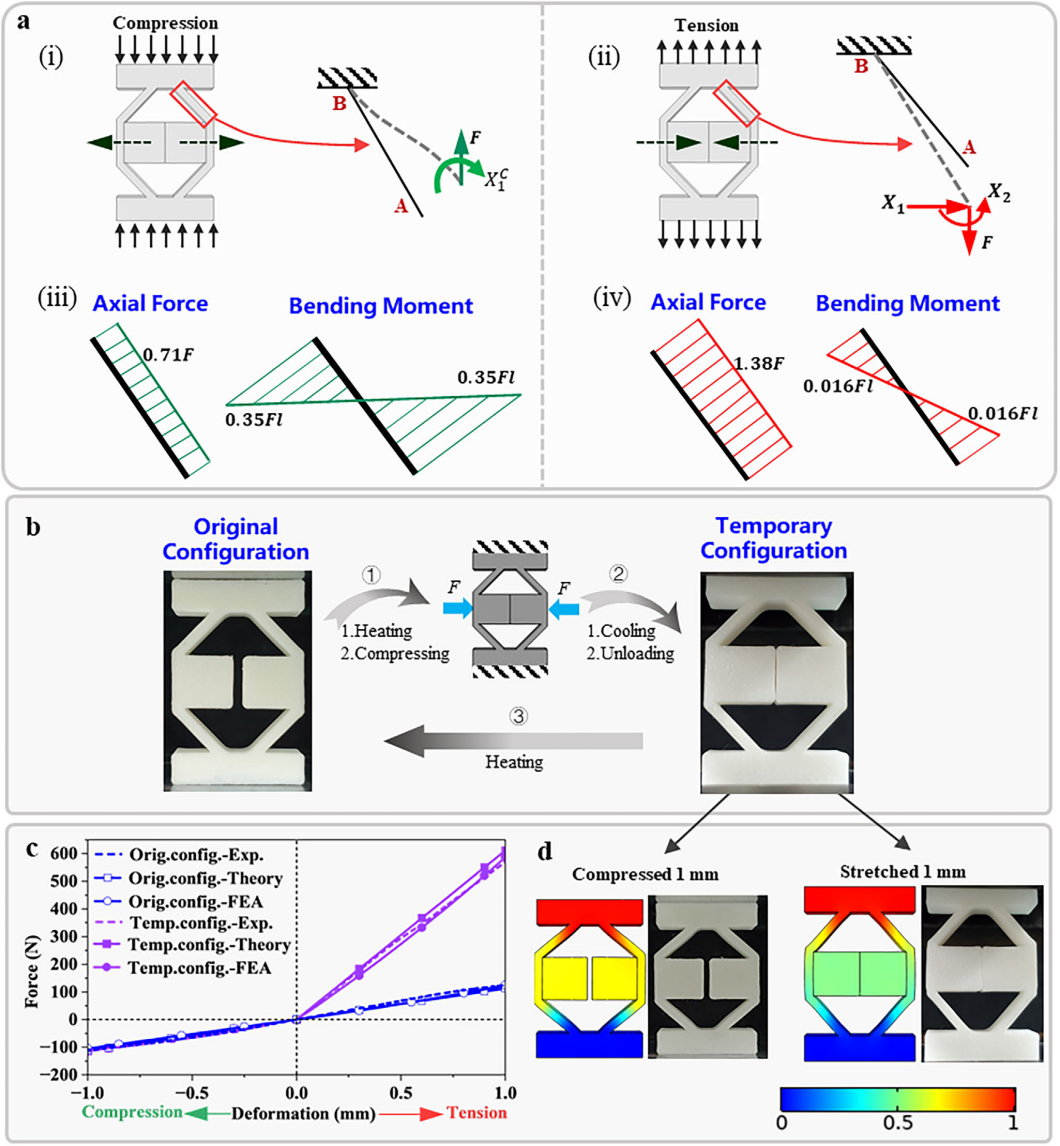
Static mechanical properties of the SVS unit cell. (a) Force diagram of the convex polygonal unit cell in the closed state: (i)during compression, the self‐contact blocks separate, and endpoint A of the diagonal beam can move laterally freely; (ii) during tension, the self‐contact blocks contact and press against each other, and endpoint A can only move vertically; (iii, iv) comparison of axial force and bending moment distributions of the diagonal beam. (b) Programming and recovery process of the convex polygonal unit cell fabricated from shape memory polymer (SMP): ① heating (60°C) and loading deformation; ② cooling and fixing followed by unloading to obtain the temporary configuration with preset contact state; ③ reheating stimulation to recover to the original configuration. (c) Comparison of force‐displacement curves between the original configuration and temporary configuration of the convex polygonal unit cell. The temporary configuration exhibits significant “stiff in tension, soft in compression” characteristics: its tensile stiffness ($k_{t}^{C}$= 594.81 N/mm) is approximately 4.5 times its compressive stiffness ($k_{c}^{C}=$130.65 N/mm), forming a sharp contrast with the tension/compression stiffness of the original configuration (∼137 N/mm). (d) Comparison of deformation modes of the temporary configuration under tension and compression: under tension, the self‐contact blocks contact and press against each other, forming an effective lateral constraint with high tensile stiffness; under compression, the self‐contact blocks separate, the lateral constraint disappears, and the compressive stiffness is low. This intuitively confirms the variable stiffness mechanism based on contact state switching.

This section establishes a theoretical model to reveal the deformation modes and variable stiffness mechanisms of the abovementioned metamaterial. Since the deformation of the metamaterial unit cell is dominated by inclined beams and exhibits symmetry, a single‐side inclined beam from the unit cell is thus selected for analysis. Taking the inclined beam of the convex polygonal unit cell (Figure 2a) as an example, the force state of the inclined beam under different loading conditions are analyzed in detail. As shown in Figure 2a(i), under compressive loading, the self‐contact blocks remain separated, allowing free in‐plane movement of endpoint A. Due to structural symmetry, the rotation angle of endpoint A remains zero (i.e., θ_*A*_ =  0) (Figure S2a). Under tensile loading, self‐contact blocks constrain each other (Figure 2a(ii)), restricting horizontal movement of endpoint A and allowing only vertical displacement (i.e., Δ_*Ax*_ = 0) (Figure S2b), while rotational symmetry maintains θ_*A*_ = 0. Given a known vertical load *F*, the canonical equations of the force method are used to solve for unknown forces, enabling calculation of axial force, bending moment, and deformation of the inclined beam. Figure S2 presents the actual force diagrams of the inclined beam under compression/tension and the unit load diagrams required for calculations.

Under compressive loading, according to the displacement compatibility condition θ_*A*_ = 0, the canonical equation is derived as

(1) $$\delta_{11}\cdot X_{1}^{C}+\Delta_{1F}=0$$

where $X_{1}^{C}$ is the unknown force at endpoint A of the inclined beam. Coefficients δ_11_ and Δ_1*F*_ are obtained via Mohr's integral:

(2) $$\delta_{11}=\int_{0}^{l}\frac{\bar{M_{1}}\left(x\right)\bar{M_{1}}\left(x\right)}{EI}dx=\frac{{\bar{M}}^{2}l}{EI}$$

(3) $$\Delta_{1F}=\int_{0}^{l}\frac{\bar{M_{1}}\left(x\right)M_{1}\left(x\right)}{EI}dx=-\frac{\sqrt{2}Fl^{2}}{4EI}$$

Here, $\bar{M_{1}}\left(x\right)$ and *M*_1_(*x*) represent the bending moment under unit moment $\bar{M}$ and load *F_c_*, respectively (Equations S1 and S2); *EI* is the bending stiffness of the inclined beam, and *l* is its length. Substituting Equations (2)‐(3) into Equation (1) yields:

(4) $$X_{1}^{C}=\frac{\sqrt{2}}{4}Fl$$

Using Mohr's integral, the vertical displacement $\Delta_{Ay}^{C}$ of endpoint A is calculated as:

(5) $$\Delta_{Ay}^{C}=\int_{0}^{l}\frac{\bar{M}\left(x\right)M\left(x\right)}{EI}dx+\int_{0}^{l}\frac{{\bar{F}}_{N}F_{N}}{EA}dx=\left(\frac{l^{3}}{12EI}+\frac{l}{2EA}\right)F$$

where ${\bar{F}}_{N}$ and $\bar{M}\left(x\right)$ are the axial force and bending moment under unit load ${\bar{F}}_{c}$ (Equations S3 and S4), while *F_N_* and *M*(*x*) represent the axial force and bending moment under actual loads *F_c_* and $X_{1}^{C}$ (Equations S5 and S6), respectively, *A* is the cross‐sectional area of the inclined beam.

For tensile loading conditions, the boundary conditions Δ_*Ax*_ = 0 and θ_*A*_ = 0 yield the canonical equations:

(6) $$\left\{\begin{array}{c} \delta_{11}\cdot X_{1}+\delta_{12}\cdot X_{2}+\Delta_{1F}=0\\ \delta_{21}\cdot X_{1}+\delta_{22}\cdot X_{2}+\Delta_{2F}=0 \end{array}\right.$$

where *X*_1_ and *X*_2_ are the unknown forces at endpoint A, their corresponding coefficients can be calculated via Mohr's integral:

(7) $$\delta_{11}=\int_{0}^{l}\frac{\bar{M_{2}}\left(x\right)\bar{M_{2}}\left(x\right)}{EI}dx+\int_{0}^{l}\frac{{\bar{F}}_{N2}{\bar{F}}_{N2}}{EA}dx=\frac{l^{3}}{6EI}+\frac{l}{2EA}$$

(8) $$\delta_{22}=\int_{0}^{l}\frac{\bar{M_{3}}\left(x\right)\bar{M_{3}}\left(x\right)}{EI}dx=\frac{l}{EI}$$

(9) $$\delta_{12}=\delta_{21}=\int_{0}^{l}\frac{\bar{M_{2}}\left(x\right)\bar{M_{3}}\left(x\right)}{EI}dx=\frac{\sqrt{2}l^{2}}{4EI}$$

(10) $$\Delta_{1F}=\int_{0}^{l}\frac{\bar{M_{2}}\left(x\right)M_{2}\left(x\right)}{EI}dx+\int_{0}^{l}\frac{{\bar{F}}_{N2}{\bar{F}}_{N2}}{EA}dx=\frac{Fl}{2EA}-\frac{Fl^{3}}{6EI}$$

(11) $$\Delta_{2F}=\int_{0}^{l}\frac{\bar{M_{3}}\left(x\right)M_{2}\left(x\right)}{EI}dx=-\frac{\sqrt{2}Fl^{2}}{4EI}$$

among them, ${\bar{F}}_{N2}$ and $\bar{M_{2}}\left(x\right)$ are the axial force and bending moment of the inclined beam under the unit load ${\bar{F}}_{x}$ (Equations S7 and S8), $\bar{M_{3}}\left(x\right)$ is the bending moment of the inclined beam under the unit moment $\bar{M}$ (Equation S9), and *F*_*N*2_ and *M*_2_(*x*) are the axial force and bending moment of the inclined beam under the load *F_t_* (EquationsS10 and S11). Substituting Equations (7)–(11) and the parameters (*l*, *E*, *I*, *A*) into Equation (6) and solving gives:

(12) $$\begin{array}{cc} X_{1}=0.952F, & X_{2}=0.163F \end{array}$$

where *X*_1_ is in N and *X*_2_ in N·mm; the coefficient 0.163 in *X*_2_ already incorporates the length dimension of the present example.

Similarly, the vertical displacement Δ_*Ay*_ of endpoint A is calculated via Mohr's integral:

(13) $$\Delta_{Ay}=\int_{0}^{l}\frac{\bar{M^{t}}\left(x\right)M^{t}\left(x\right)}{EI}dx+\int_{0}^{l}\frac{{\bar{F}}_{N}^{t}{\bar{F}}_{N}^{t}}{EA}dx=kF$$

among them, ${\bar{F}}_{N}^{t}$ and $\bar{M^{t}}\left(x\right)$ are the axial force and bending moment equations of the beam under the unit load ${\bar{F}}_{t}$ (Equations S12 and S13), and $F_{N}^{t}$ and *M^t^* (*x*) are the axial force and bending moment equations of the beam under the actual loads *F_t_*, *X*_1_, and *X*_2_ (Equations S14 and S15). Substituting the parameters of this example gives *k*  =  0.0016 mm/N.

Based on the superposition principle, the axial force and bending moment distributions across arbitrary cross‐sections of the inclined beam can be calculated. As shown in Figure 2a(iii), under compression conditions, the bending moment at section A of the inclined beam is *M*  =  0.35*Fl*. In comparison, under tensile conditions (Figure 2a(iv)), the bending moment at this section significantly decreases to *M*  =  0.017*Fl*, representing a reduction of approximately 95% compared to the compression condition, indicating a significant weakening of the bending effect. The axial force at section A under compression conditions is 0.71*F* while under tensile conditions, the axial force increases to 1.35*F*, representing a relative increase of approximately 90%. This clear evolutionary trend revealed by the comparison indicates that under compressive loads, the mechanical response of the structure is dominated by bending moments, corresponding to a bending‐dominated deformation mode; whereas under tensile loads, it transforms to a tension‐dominated deformation mode dictated by axial forces. Studies have shown that tension‐dominated structures exhibit higher specific stiffness compared to bending‐dominated structures [47].

To enable reconfiguration, shape memory polymer (SMP4D BASIC 3D printing filament, Shenzhen Xingyi Technology Co., China) was selected as the matrix material (the specific composition is undisclosed due to intellectual property restrictions). Taking the Convex Unit Cell as an example, the reconfiguration process involves three stages (Figure 2b): ① Heating and deforming: the original metamaterial unit was heated to 60°C and deformed under load; ② Cooling and fixing: the deformed unit was cooled to room temperature under maintained load, then unloaded to obtain a stable temporary configuration; ③ Stimulus recovery: reheating to 60°C triggered recovery from the temporary configuration to the original shape (see Movie S1). It should be noted that, if specific self‐contact block spacing needs to be preset in the temporary configuration, it is only necessary to insert a spacer block with a thickness of δ between the self‐contact blocks before deformation in step ①, and remove it after completing step ②.

To validate the theoretical model, finite element analysis (FEA) and experimental tests were conducted to verify the mechanical properties of metamaterial unit cells. Figure 2c presents the force‐deformation curves of the convex polygonal unit cell in its original and temporary configurations. Good agreement is observed between FEA, experimental, and theoretical results, confirming model accuracy. Notably, the temporary configuration (purple curve) exhibits significantly higher tensile stiffness ($k_{t}^{C}=594.81\;N/\mathrm{mm}$) compared to that under other three loading conditions (original tension/compression and temporary compression), all of which show comparable stiffness values (130.65–137.55 N/mm). This corresponds to a stiffness enhancement by a factor of approximately 4.5 under tension in the temporary configuration. Furthermore, the compressive stiffness $k_{c}^{C}$ of the temporary configuration is 130.65 N/mm, which is much lower than its tensile stiffness $k_{t}^{C}$. This indicates that the temporary configuration of the Convex Unit Cell exhibits an asymmetric characteristic of “high tensile stiffness and low compressive stiffness”, i.e., “stiff in tension, soft in compression” (Figure 2d shows its deformation diagram). In contrast, the re‐entrant polygonal unit cell exhibits the asymmetric characteristic of “soft in tension, stiff in compression” (see details in Section S3), which complements the characteristic of the convex polygonal unit cell.

## 4 Reconfigurable SVS Metamaterials for Information Storage and Encryption

This section constructs a metamaterial capable of information storage and encryption by arranging the proposed reconfigurable SVS metamaterial unit cells in an in‐plane array. By adjusting the distance separating the self‐contact blocks within each constituent unit cell, the stiffness of that unit is modulated. This causes a pre‐designed pressure distribution to appear on the back surface of the metamaterial under an applied load, thereby illuminating the correspondingly connected LEDs to achieve information storage and retrieval. Furthermore, by varying the distances of the self‐contact blocks among different unit cells, the temporal sequence of stiffness change for each unit cell can be programmed. This capability allows for the implementation of information encryption. Taking the Re‐entrant polygonal unit cell as a case study, this section validates the feasibility of the proposed method for information storage and encryption through a combination of finite element simulations and experimental tests.

### 4.1 Information Storage

**FIGURE 3:**
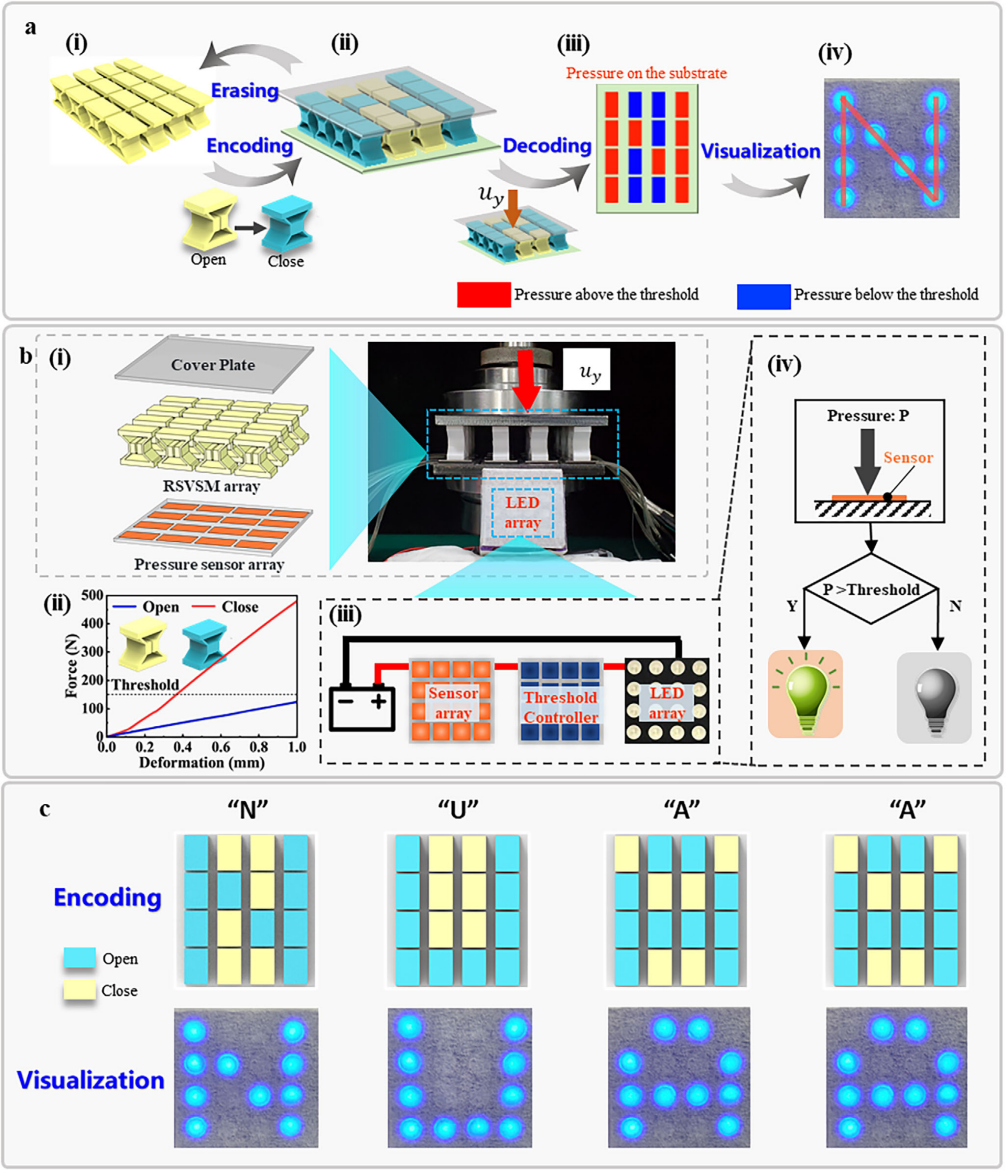
Information storage using an in‐plane array metamaterial based on re‐entrant polygonal unit cells. (a) Information storage process: (i) initial state of the 4 × 4 in‐plane array metamaterial; (ii) programming and writing the information to be stored (e.g., the letter “N”) into the array by reconfiguring specific unit cells from the initial (“open”, low‐stiffness) state to the temporary (“closed”, high‐stiffness) state; (iii) applying pressure *u_y_* to the in‐plane array metamaterial, the back of the metamaterial exhibits different pressure distributions, with red and blue indicating pressures above and below the detection threshold, respectively; (iv) an external LED array is used, where LEDs corresponding to pressures exceeding the threshold are illuminated, achieving the visual readout of the stored information “N”. (b) Information reading device and principle: (i) a thin‐film pressure sensor array is attached to the back of the array metamaterial; (ii) the sensor trigger threshold (*F_T_* =  150N) is determined from the force‐displacement curves of the metamaterial unit in the “open” and “closed” states; (iii) connection principle between the pressure sensor array and the LED array; (iv) information reading principle: when the pressure on a corresponding thin‐film pressure sensor exceeds the threshold, its LED light illuminates, finally displaying the corresponding stored information in the LED array. (c) Sequential storage of letters ‘N’, ‘U’, and ‘A’ using the same 4 × 4 in‐plane array metamaterial.

Figure 3a(i) shows a 4 × 4 in‐plane array structure based on re‐entrant polygonal unit cells. Each unit cell in the array exhibits independently reconfigurable properties, enabling active regulation of the stiffness distribution within the array. The regulation mechanism lies in that each unit cell serves as an independent pressure transmission path, whose force transmission capability is directly related to its own stiffness state. When subjected to the same compressive force and undergoing the same deformation, units in the low‐stiffness state (i.e., “open” state) transmit less pressure to the back of the metamaterial, while units in the high‐stiffness state (i.e., “closed” state) transmit greater pressure to the back of the metamaterial. Based on this mechanism, by programming the stiffness distribution of the metamaterial array (Figure 3a(ii)), the pressure transmitted to the back of the metamaterial can be adjusted as needed (Figure 3a(iii)).

Based on this principle, the array metamaterial can be used for information storage. The specific method is as follows: the array metamaterial is treated as a 4 × 4 pixelated matrix (assuming all units are in their original configuration, “open” state, in the initial state). Geometric reconstruction of array units can then be regarded as binary conversion of corresponding pixels. By reconfiguring predetermined units, specific patterns can be “written” into this matrix, thereby achieving information encoding. For example, as shown in Figures 3a(ii) and S9, if the units in Column 1, Column 4, and positions (2,2) and (3,3) are reconfigured to the temporary configuration (“closed” state), the pattern formed by the high‐stiffness units in the pixel matrix corresponds to the uppercase letter “N” (Figure 3a(iii)), thus completing the encoding and storage of information.

After information “writing”, to achieve visual reading of information, a thin‐film pressure sensor array is arranged below each unit in the array (Figure 3b(i)) and connected to a 4 × 4 LED dot matrix (each sensor can only trigger the LED at its corresponding position), with the connection method shown in Figures 3b(iii) and S11b. The trigger logic of the sensor is set as follows: when the detected pressure value exceeds the preset threshold (150 N), the corresponding LED is triggered to light up; otherwise, the light remains off (Figure 3b(iv)). The preset threshold is set higher than the maximum pressure transmitted by low‐stiffness units (Figure 3b(ii)), ensuring that low‐stiffness units cannot light the corresponding LEDs under the specified displacement load (1 mm), thus achieving accurate distinction between the two types of units. Experimental results show that when a uniform compressive displacement *u_y_* is applied to the array metamaterial (Figures 3b(i) and S11c), high‐stiffness units trigger the LEDs, while the stress of low‐stiffness units does not reach the threshold and thus does not light the LEDs. This causes the LED matrix to display an “N” pattern consistent with the stored information, successfully realizing visual reading of information (Figure 3a(iv), Movie S2).

As mentioned above, the temporary configuration can automatically recover to the original configuration when heated to 60°C. Leveraging this feature, erasure of stored information (equivalent to reformatting) can be achieved by heating. After information erasure, new information can be encoded via unit reconfiguration. As shown in Figure 3c, we successively stored the letters “N”, “U”, and “A” using this in‐plane array metamaterial. Furthermore, by increasing the number of unit cells, the resolution of the pixel matrix can be effectively improved, enabling the encoding of more complex information.

### 4.2 Information Encryption

**FIGURE 4:**
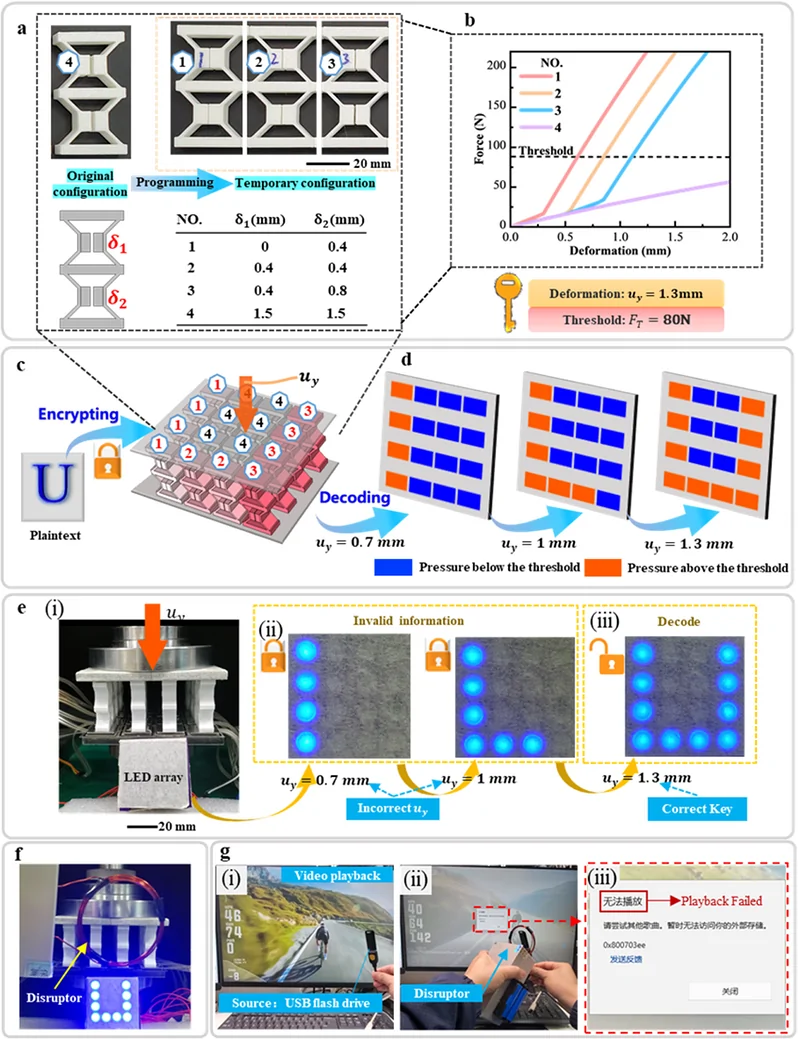
In‐plane array metamaterial for information encryption. (a) The double‐cell units are labeled as 1, 2, 3, and 4 according to the different distances of their upper and lower self‐contact blocks (i.e., δ_1_, δ_2_). (b) Force‐displacement curves of the four types of units (labeled 1, 2, 3, 4) under compression. The smaller the total gap δ (δ  = δ_1_  + δ_2_), the earlier the stiffness mutation occurs. The sensor trigger threshold (*F_T_* =  80 N) is determined based on the force‐displacement curves. (c) Encoding the encrypted information (“U”) and ambiguous information (“I”, “L”) into a 4 × 4 array. (d) Schematic diagram of the pressure distribution changes on the back of the in‐plane array metamaterial under an increasing compressive displacement *u_y_*. (e) Information encryption/decryption process: (i) photograph of the LED matrix arrangement; (ii) applying an incorrect decryption key (*u_y_* =  0.7 mm or 1 mm) activates only some of the target units, causing the LED array to display an incorrect pattern (“I” or “L”); (iii) applying the correct key (*F_T_* =  80 N, *u_y_* = 1.3 mm) simultaneously activates all target units, thereby displaying the encrypted information “U”. (f) Effect of electromagnetic interference on the proposed metamaterial: no observable effect. (g) Effect of electromagnetic interference on video playback from a USB flash drive: (i) before interference, the video is playing normally; (ii) after interference, the video fails to play; (iii) enlarged view of the error dialog, showing a message indicating that the video cannot be played.

In addition to information storage, this in‐plane array strategy can also be used for encrypted information storage. The key mechanism of information encryption stems from the precise temporal control of stiffness mutations of internal units within the array. As shown in Figure 4a–c, the metamaterial is also a 4 × 4 unit array. Its basic unit consists of two re‐entrant polygonal unit cells connected in series (Figure 4a). In the initial state, all units are in their original configuration, with the preset distances δ_1_ and δ_2_ of their self‐contact blocks being equal (both 1.5 mm), and this state is marked as “4” (to distinguish from subsequent temporary configurations with different δ values). The preset distances δ_1_ and δ_2_ of the unit can be regulated through geometric reconfiguration, and the δ value (δ  = δ_1_  + δ_2_) has a clear correlation with the timing of stiffness mutations: the smaller the δ value, the earlier the unit undergoes self‐contact stiffness mutation during compression. Therefore, by precisely setting the δ value of each unit through reconfiguration, the sequence of stiffness mutations of each unit in the array can be controlled.

To achieve specific temporal actions, we perform geometric reconfiguration on the target units (numbered 1–3) in the array structure (Figure 4c), with the values of δ_1_ and δ_2_ shown in Figure 4a. The sum of the preset distances (δ) of the self‐contact blocks for units 1 to 4 shows an increasing trend. This increasing distance design directly determines their response order under compressive load: as shown in the force‐deformation curve in Figure 4b, during compression, units 1 to 3 undergo stiffness mutations in sequence, while unit 4 remains in a low‐stiffness state throughout. When a uniform compressive displacement *u_y_* is applied to the array, units 1–3 will sequentially reach the preset threshold (Figure 4b, Threshold = 80 N) in terms of transmitted pressure when the overall compressive deformation reaches 0.7, 1, and 1.3 mm, respectively. The pressure distribution diagram on the back of the in‐plane array metamaterial intuitively illustrates this stiffness mutation order (Figure 4d). It should be noted that the double‐cell configuration, rather than single cells, is adopted here for the following consideration. Each unit consists of two re‐entrant cells connected in series, allowing the gap distances of the upper and lower cells (δ_1_ and δ_2_) to be independently adjusted. By varying the combination of these two gaps, the compression displacements required for different units to reach a given pressure threshold can be significantly differentiated with relatively coarse step sizes. As shown in Figure 4b, units 1∼4 reach the threshold at compression displacements of approximately 0.7, 1.0, 1.3, and 1.5 mm, respectively. If single cells were used instead, all four gap values would need to be set within a limited range, leaving much narrower intervals between the threshold displacements of different units. Consequently, the responses of different units would be more susceptible to overlapping due to manufacturing tolerances or loading deviations. The double‐cell configuration effectively widens the response windows of each unit, making the timing control more robust and reliable.

The spatially and temporally controllable stiffness mutation described above lays the foundation for achieving information encryption. By precisely controlling the spatial distribution of the units and the order in which their pressures reach the preset threshold, the in‐plane array metamaterial can form expected patterns, thereby enabling the encoding and storage of encrypted information. Different from abovementioned information storage, which relies only on the spatial distribution of stiffness, the encryption metamaterial adds a temporal dimension. It requires matching both the spatial position of units and the timing of stiffness mutations for decryption, thus achieving encryption. The implementation involves several steps. First, the target units in the array metamaterial are reconfigured to complete the encoding of the information to be encrypted, with ambiguous information introduced during encoding to enhance security. A specific displacement load *u_y_* and a sensor threshold *F_T_* are set as the decryption key. By applying the specified displacement load *u_y_* to the in‐plane array metamaterial, certain positions in the LED matrix are triggered to reach the threshold, achieving decryption. If the threshold *F_T_* or the displacement *u_y_* is set incorrectly, the LED matrix will display ambiguous information, and decryption will fail. Taking the information to be encrypted, “U,” as an example, units 1–4 are arranged into a 4 × 4 array as shown in Figure 4c. The image formed by units 1, 2, and 3 is “U,” while unit 1 alone forms ambiguous information “I,” and units 1 and 2 together form ambiguous information “L.” Based on the force‐displacement curves of each unit (Figure 4b), the pressure threshold *F_T_* = 80 N and the displacement load $u_{y}\in \left[1.2,1.5\right]\mathrm{mm}$ are set as the decryption key. The ingenuity of this key lies in that only when *u_y_* is within this range will the pressures of all units constituting “U” (units 1, 2, and 3) exceed the 80N threshold. If *u_y_* deviates from this range, either an insufficient number of units are triggered, or non‐target units are triggered, making it impossible to form valid information.

Experiments for encrypted information storage were conducted, and the results are shown in Figure 4e. It can be observed that when the correct decryption key is applied, the pressures of the corresponding target units reach the threshold *F_T_* = 80 N, the corresponding LEDs light up, and the LED matrix clearly displays letter “U” (Figure 4e(iii)). When the key is incorrect, ambiguous information is output. As shown in Figure 4e(ii), these are the display results (“I”/“L”) when the displacement load (*u_y_* =  0.7 mm/1 mm) is incorrect, both cases resulting from an insufficient number of target units being triggered (see details in Movie S3). Similarly, if the threshold is set incorrectly, invalid information will also be displayed. For instance, as shown in Figure S12, this is the display result (“L”) when the threshold (130N) is incorrect.

Furthermore, we can roughly estimate the information storage density based on the self‐contact variable stiffness metamaterial. Taking the case where each letter is represented by a 4 × 4 in‐plane array as an example, and assuming the top and bottom surface side lengths of the metamaterial unit are *a* and *b* respectively (Figure 4d), the information storage density of the proposed scheme can be calculated as:

(14) $$\phi_{s}=\frac{1\mathrm{Byte}}{4a\times 4b}$$

Considering that current metamaterial fabrication employs 3D printing, with a known minimum feature size of approximately 0.1 mm, the minimum values of *a* and *b* are 0.1 and 1.31 mm, respectively (the detailed derivation is provided in Section S4). The upper limit for the information storage density is thus about 465.9 KB/m^2^. It should be pointed out that the storage density based on this metamaterial still has a considerable gap compared to that of current conventional chips. This is primarily limited by the precision of current 3D printing. If advanced micro/nano‐fabrication methods were used to improve the structural manufacturing precision, the information storage density of the metamaterial would also be significantly enhanced. It is worth emphasizing that the advantage of using mechanical metamaterials for storage and encryption lies not in storage density, but rather in providing an information processing method capable of functioning in complex and extreme environments, such as strong magnetic fields. Traditional electronic storage devices are prone to failure in such conditions, whereas mechanical metamaterials exhibit high robustness.

Beyond static storage density, evaluation of the information writing/rewriting speed is also performed. The information writing process relies on the shape memory effect and consists of three sequential steps: (i) heating the unit above the glass transition temperature to enter the deformable rubbery state (∼15 s); (ii) applying mechanical loading to program the target temporary configuration (∼5 s); and (iii) cooling to room temperature to fix the temporary shape (∼5 s). A complete write cycle therefore takes approximately 25 s, corresponding to a refreshing frequency of 0.04 Hz. Since erasing is achieved simply by heating, and heating is itself the first step of the writing process, no additional operation is thus required for erasure. Rewriting is exactly the same as the writing cycle. It should be noted that this write speed is orders of magnitude slower than electronic memories. However, for archival‐type storage in extreme environments where electronic storage fails (e.g., strong electromagnetic interference, nuclear radiation), write times on the order of tens of seconds to minutes are acceptable.

To validate the reliability of the proposed mechanical metamaterial in complex electromagnetic environments, a comparative electromagnetic interference (EMI) experiment was further conducted. As shown in Figure 4f,g, a conventional electronic storage device (using a USB flash drive as an example) and the proposed metamaterial were placed in the same electromagnetic field. The experimental results indicated that after exposure to EMI, the video file stored on the conventional electronic storage device could not be accessed, resulting in playback failure. (Figure 4g(ii) and Movie S4). In contrast, as a purely mechanical physical information platform, the information storage and readout processes of the metamaterial depend entirely on its stiffness distribution and mechanical response, granting it intrinsic immunity to external electromagnetic fields. As illustrated in Figure 4f, after exposure to the same EMI, the mechanical response behavior and the corresponding readout pattern of the LED array remained unchanged (Movie S5), demonstrating that the stored information remained intact. These results demonstrate the superior reliability and robustness of the proposed metamaterial.

## 5 Reconfigurable SVS Metamaterials for Information Transmission

The reconfigurable SVS metamaterial can also be utilized for directional information transmission. As mentioned above, in the field of information transmission, a substance itself can serve as an information carrier, with information being conveyed by controlling the movement of that substance. In this section, by integrating re‐entrant polygonal unit cells (“soft in tension, stiff in compression”) with Convex Unit Cells (“stiff in tension, soft in compression”), we propose a metamaterial for information transmission that features asymmetric stiffness and reversible switching of this asymmetry. This programmable stiffness asymmetry enables the metamaterial to convert symmetric external excitations into directionally controllable, asymmetric vibration responses, which in turn generates a controllable driving force. This achieves the directional transport of substances such as droplets and mass blocks, while simultaneously allowing the transport direction to be adjusted on demand.

### 5.1 Programmable Switching of Asymmetric Stiffness

As mentioned in section 3, SVS metamaterials naturally exhibit asymmetric stiffness features. Furthermore, re‐entrant polygonal unit cells (exhibiting “soft in tension, stiff in compression” characteristics) and convex polygonal unit cells (exhibiting “stiff in tension, soft in compression” characteristics) possess natural functional complementarity in their asymmetric stiffness responses. Therefore, this section further combines these two types of unit cells and introduces a “dormant/active” control mechanism to achieve active switching between the two asymmetric stiffness modes. Specifically, when the re‐entrant polygonal unit cells are in the active state (closed, high compressive stiffness) and the convex polygonal unit cells are in the dormant state (open, low compressive and tensile stiffness), the metamaterial exhibits “soft in tension, stiff in compression” characteristics. Conversely, if the re‐entrant polygonal unit cells are switched to the dormant state (open, low compressive and tensile stiffness) and the convex polygonal unit cells are in the active state (closed, high tensile stiffness), then the metamaterial as a whole exhibits “ stiff in tension, soft in compression” characteristics. Therefore, through geometric reconfiguration, precise control over the dormant/active states of the two types of unit cells allows for active regulation of the metamaterial's asymmetric stiffness behavior.

**FIGURE 5:**
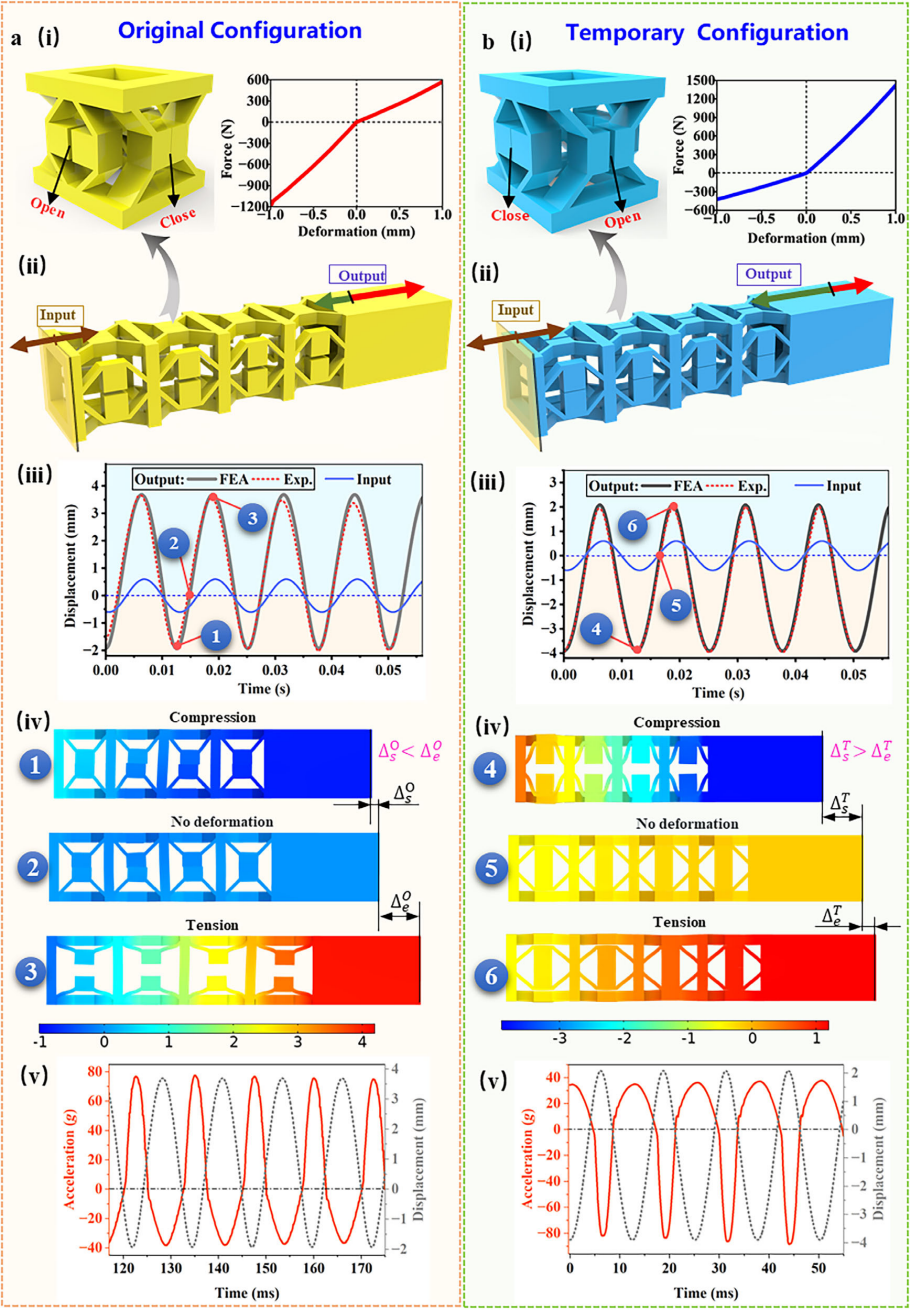
Asymmetric vibration characteristics of the reconfigurable SVS metamaterial. (a) Original configuration of the metamaterial: (i) the four‐cell metamaterial unit and its force‐displacement curve, exhibiting “soft in tension, stiff in compression” asymmetric characteristics; (ii) schematic diagram of the metamaterial, composed of four identical four‐cell units connected in series; (iii) comparison of dynamic response curves for the input end (left side, with harmonic excitation input: amplitude 0.6 mm, frequency 80 Hz) and the output end (right side) obtained from experimental test and simulation analysis. The output end exhibits asymmetric vibration where the forward amplitude is greater than the negative amplitude (i.e., forward amplitude $\Delta_{e}^{O}$= 3.7 mm, negative amplitude $\Delta_{s}^{O}$= 1.8 mm); (iv) deformation at three typical vibration moments (corresponding to points ①, ②, ③ in (iii)). At moment ①, the metamaterial has a small negative displacement; at moment ③, the metamaterial has a relatively large forward displacement; (v) Acceleration response at the output end. (b) Temporary configuration after reconfiguration: (i) the four‐cell metamaterial unit and its force‐displacement curve, exhibiting “stiff in tension, soft in compression” asymmetric characteristics; (ii) schematic diagram of the reconfigured metamaterial; (iii)comparison of dynamic response curves for the input end (left side, with harmonic excitation input: amplitude 0.6 mm, frequency 80 Hz) and the output end (right side) obtained from experimental test and simulation analysis. The output end exhibits asymmetric vibration where the negative amplitude is greater than the forward amplitude (i.e., negative amplitude $\Delta_{s}^{T}$= 3.9 mm, forward amplitude $\Delta_{e}^{T}$= 2 mm); (iv) deformation at three typical vibration moments (corresponding to points ④, ⑤, ⑥ in (iii)). At moment ⑥, the metamaterial has a small forward displacement; at moment ④, the metamaterial has a relatively larger negative displacement; (v) Acceleration response at the output end.

To verify the switchability of the metamaterial's asymmetric stiffness characteristics, a four‐cell metamaterial composed of two closed re‐entrant polygonal unit cells and two open convex polygonal unit cells is designed. As shown in Figure 5a(i), the four unit cells are arranged in a rectangularly symmetric distribution, with the two pairs of opposite sides being formed by different unit cells, forming a compact and axisymmetric original configuration. Through geometric reconfiguration, this original configuration can be transformed into a temporary configuration (Figure 5b(i)). After reconfiguration, the open/closed states of the unit cells are reversed: the re‐entrant polygonal unit cells switch from a closed state to an open state, while the convex polygonal unit cells switch from an open state to a closed state. Experimental results show that the original configuration exhibits “soft in tension, stiff in compression” characteristics, with its compressive stiffness (1239 N/mm) being significantly higher than its tensile stiffness (511 N/mm). After being reconfigured into the temporary configuration, this asymmetry is completely reversed, exhibiting “stiff in tension, soft in compression” characteristics, with its tensile stiffness (1283 N/mm) being significantly greater than its compressive stiffness (456 N/mm). This switch of asymmetric stiffness stems from the reversal of self contact states caused by structural reconfiguration. In the original configuration, the self contact blocks in the re‐entrant polygonal unit cells are activated during compression to form constraints, while all self contact blocks are dormant during tension, thus exhibiting “soft in tension, stiff in compression” (see deformation details in Figure S13a). In the temporary configuration, the activation and dormant pattern of the self contact blocks is completely opposite: the self‐contact blocks in the convex polygonal unit cells are activated during tension, while all self contact blocks are in a dormant state during compression. Thus, the macroscopic characteristics are reversed to “stiff in tension, soft in compression” (see deformation details in Figure S13b). Therefore, through geometric reconfiguration, active control of the direction of stiffness asymmetry can be achieved.

### 5.2 Asymmetric Vibration Characteristics Induced by Asymmetric Stiffness

The asymmetric stiffness characteristics of the SVS metamaterial enable the conversion of symmetric vibration into asymmetric motion, a characteristic that lays the foundation for the directional control of mechanical energy, for instance, achieving the directional transport of substances like mass blocks or droplets. Based on this concept, we construct a metamaterial for directional substance transport (Figure 5a(ii),b(ii)) using the programmable unit cells with switchable asymmetric stiffness shown in Figure 5a(i),b(i). Under external excitation, this directional transport metamaterial can utilize its own asymmetric stiffness to generate asymmetric vibrations (Figure 5a(iii),b(iii)). Furthermore, the metamaterial can switch the direction of its stiffness asymmetry (e.g., from “stiff‐in‐tension and soft‐in‐compression” to “soft‐in‐tension and stiff‐in‐compression”) through geometric reconfiguration, thereby reversely controlling the direction of the substance transport. According to the principle of stick‐slip actuation, the asymmetric vibration of the structure can be used for the directional transport of objects attached to it: when the structure's vibration displacement amplitude is large, its acceleration is small, and the resulting inertial force fails to overcome the maximum static friction between the attached object and the structure. The object and the structure remain ‘stuck’ together due to static friction and thus move in unison. Conversely, when the vibration displacement amplitude is small, the acceleration is large, and the inertial force overcomes the static friction, causing the attached object to ‘slip’ relative to the structure (i.e., producing a small displacement difference). Through this cyclic ‘stick‐slip’ action, the attached object moves in a specific direction [48, 49]. Additionally, studies have shown that when a droplet is placed on the non‐smooth surface of an asymmetrically vibrating structure, the net inertial force acting on the droplet causes it to move directionally. This droplet manipulation method of droplet manipulation based on asymmetric vibration not only has significant application potential in fields such as biomedical detection, chemical microreactions, and precise infusion therapy [50], but can also be used to construct fluidic logic gates or microfluidic systems to transmit computational and logical information, such as by defining the “presence/absence” or “arrival/non‐arrival” of a droplet as a binary “1/0” to realize logical operation functions. Therefore, this directional transport metamaterial also lays the foundation for information transmission.

To verify the substance transport ability of the metamaterial, we conducted both experiments and finite element simulations in this section. First, the dynamic response of the metamaterial is analyzed numerically to provide a theoretical basis for the subsequent directional transport experiments. For the original configuration, when a symmetric vibration (amplitude of 0.6 mm and frequency of 80 Hz) is applied to the left side of the metamaterial, the vibration of the right platform exhibits significant asymmetry, with a forward amplitude (3.7 mm) greater than its negative amplitude (1.8 mm) (Figure 5a(iii)). The small error between the experimental tests and the finite element analysis results demonstrates the validity of the simulation model. Subsequently, the finite element simulation model is used to analyze the deformation diagrams of the metamaterial at typical moments (points ① & ④: compressed to maximum displacement, points ② & ⑤: at equilibrium position, points ③ & ⑥: stretched to maximum displacement) (Figures 5a(iv), b(iv) and S15a,b), revealing its asymmetric vibration mechanism. As shown in Figure 5a(iv), during the compression phase (point ①), the self‐contact blocks of re‐entrant polygonal unit cells are closed. This contact constraint significantly increases the compressive stiffness of the metamaterial, thereby limiting the negative amplitude (i.e., $\Delta_{s}^{O}$ is small); whereas during the tension phase (point ③), the self‐contact blocks are separated, and both the tensile and compressive stiffness of the metamaterial are low, leading to a significantly larger forward amplitude (i.e., $\Delta_{e}^{O}$ is large). Similarly, the temporary configuration, when the same harmonic excitation is applied to the left side of the metamaterial, still exhibits significant asymmetric vibration, with a forward amplitude $\Delta_{e}^{T}$ of 2 mm and a negative amplitude $\Delta_{s}^{T}$ of 3.9 mm (Figure 5b(iii)). However, due to the switched direction of stiffness asymmetry, the temporary configuration displays the opposite pattern to the original configuration: the negative amplitude is now larger than the forward amplitude.

Since the displacement responses are asymmetric, the acceleration responses are necessarily asymmetric as well. Figure 5a(v),b(v) present the acceleration response curves at the output end for the original and temporary configurations, respectively. For the original configuration, the forward acceleration amplitude is approximately 78 G_0_ and the negative acceleration amplitude is approximately 38 G_0_, (where G_0_ is the gravitational acceleration), with the forward acceleration being significantly larger than the negative. This is because the original configuration exhibits higher stiffness in compression (“stiff in compression”) and lower stiffness in tension (“soft in tension”). During compression, the restoring force and acceleration are larger, while during tension they are smaller, resulting in a larger forward (compression‐direction) acceleration amplitude than the negative (tension‐direction) amplitude. For the temporary configuration, the direction of stiffness asymmetry is completely reversed: the forward acceleration amplitude is approximately 37 G_0_ and the negative acceleration amplitude is approximately 81 G_0_, with the negative acceleration being significantly larger than the forward. These acceleration responses are fully consistent with the asymmetry observed in the displacement responses, further confirming the existence of asymmetric vibration. It is this asymmetric vibration that provides the driving force for directional matter transport. Detailed results and discussion of the frequency sweep, resonance characterization, and damping analyses are provided in Section S5.

### 5.3 Functional Validation of Directional Transport

This subsection utilizes the aforementioned asymmetric vibration characteristics of the metamaterial to conduct experimental verification of directional substance transport. First, leveraging the asymmetric vibration generated by the metamaterial, we verified the directional actuation of droplets.

**FIGURE 6:**
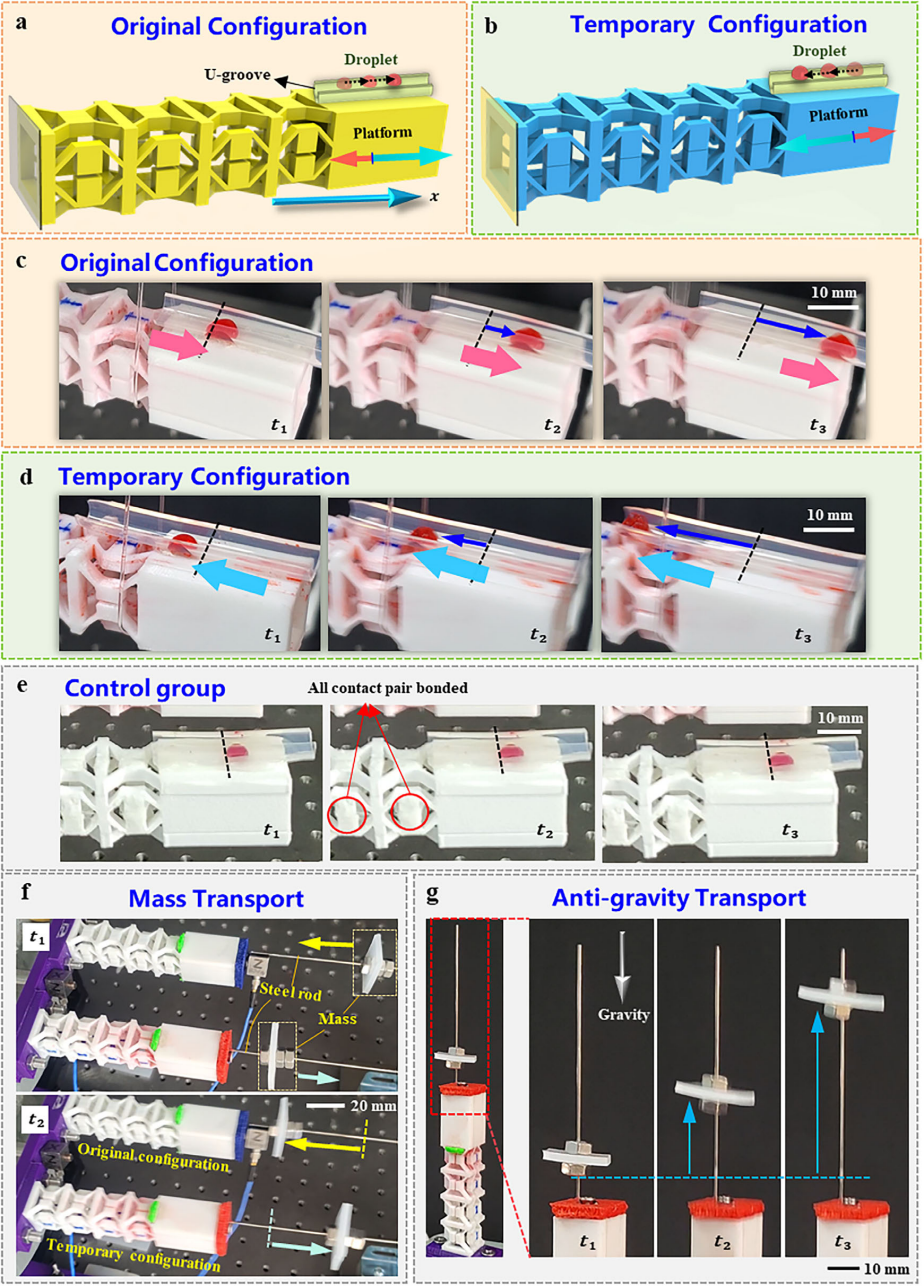
Substance transport experiments of the reconfigurable SVS metamaterial. (a, b) Schematic diagrams of the droplet transport experimental setup for the (a) original and (b) temporary configurations of the metamaterial. A silicone U‐shaped groove is fixed to the output platform of the metamaterial to carry the droplet. (c, d) Sequential experimental images of the directional motion of a droplet within the U‐shaped groove under an 80 Hz harmonic excitation for the (c) original and (d) temporary configurations of the metamaterial (see Movie S6 for details), (*t*_1_ =  0 ms,  *t*_2_ = 700 ms,  *t*_3_ = 1400 ms). (e) Sequential experimental images of droplet motion within the U‐shaped groove for the control experiment, where the self‐contact blocks were glued and fixed (see Movie S7 for details). (f) Schematic diagram of the mass block transport experimental setup for the metamaterial, with the mass block threaded onto a stainless‐steel guide rod. Comparison of the motion directions of the mass block connected to the original and temporary configurations under horizontal excitation. (g) When the metamaterial is placed vertically, the mass block connected to the temporary configuration achieves anti‐gravity upward motion (see Movie S8 for details).

As shown in Figure 6a,b, a silicone U‐shaped groove (with a cross‐sectional width of 6 mm and a radius of 3 mm) was fixed to the output platform of the metamaterial, causing it to undergo asymmetric vibrations along with the platform. In the experiment, a droplet was placed at the midpoint of the U‐shaped groove, and a harmonic excitation with a frequency of 80 Hz was applied to the base of the metamaterial, which effectively drove the droplet within the groove to move directionally. The experimental results show that with the original configuration, the U‐shaped groove generates forward‐dominant vibrations, and the droplet moves to the right along the groove (Figure 6c); whereas when reconfigured to the temporary configuration, the metamaterial produces negative‐dominant vibrations, and the droplet moves to the left (Figure 6d). Furthermore, to demonstrate the robustness of the metamaterial for droplet transport, we tested two experimental operation sequences. Regardless of the sequence of placing the droplet in the U‐shaped groove and the application of the vibration excitation, the droplet is stably transported in a unidirectional manner (see details in Movie S6). Figure 6e shows the droplet transport result for the control group, i.e., a metamaterial where all self‐contact blocks are glued and fixed. The experiment indicates that because the relative motion of the self‐contact blocks is completely constrained, the structure does not possess stiffness asymmetry or vibration directionality. The droplet only oscillates back and forth near its initial position with no movement relative to the U‐shaped groove (see details in Movie S7). This result confirms that stiffness modulation based on self‐contact is the key to achieving directional droplet transport. In addition, the directional transport function of this metamaterial can be further extended. For example, by orthogonally arranging metamaterials, two‐dimensional droplet sorting can be achieved, manipulating droplets into designated channels (Figure S20). It should be noted that programmable control over the droplet's path essentially provides a mechanical microfluidic logic operation unit. For instance, the choice of a left or right path can be directly defined as executing a logical instruction of ‘0’ or ‘1’. This lays the foundation for constructing passive microfluidic and droplet routing systems that do not rely on external pumps and valves, and holds promise for achieving complex and diverse information transmission.

Furthermore, to further validate the directional substance transport performance of the metamaterial, we also conducted directional transport experiments using a mass block. The experimental setup is shown in Figure 6f, where a thin, long stainless‐steel rod was fixed to the output platform of the metamaterial, causing it to undergo asymmetric vibrations with the platform. A mass block was threaded onto the stainless‐steel guide rod (Figure S21). The experiments show that when a harmonic excitation is applied to the horizontally placed metamaterial, the mass block moves to the left for the original configuration, while for the temporary configuration, it moves to the right (Figure 6f). As shown in Figure 6g, if the metamaterial is placed vertically (with the excitation end at the bottom), the mass block on the temporary configuration can perform anti‐gravity motion, i.e., moving continuously upward (see details in Movie S8). This driving method does not require complex transmission mechanisms, providing a new route for applications such as precision positioning and diverse information transmission.

It should be emphasized that the current speed of directional transport achieved by the metamaterial is approximately 30 mm/s. This is primarily limited by the combined effect of the driving force and the friction on the platform surface; increasing the driving force and reducing platform friction would significantly enhance the transport speed. However, it is difficult for the transmission speed of mechanical media to match that of current electronic signals. Therefore, as discussed in the preceding Section 4, the directional transport method proposed in this paper is not intended to replace existing electronic information transmission methods. Instead, it provides a viable and robust complementary solution for information transmission in extreme environments, such as those with electromagnetic interference, strong radiation, or high pressure.

## 6 Conclusions

This study presented the design and application of a reconfigurable SVS mechanical metamaterial based on SMPs. By integrating the shape memory with the SVS mechanisms, this work facilitated the regulation and reversible programming of structural stiffness and its tension‐compression asymmetry, thereby transforming the mechanical metamaterial from passive load‐bearing components into intelligent physical platforms for information processing and manipulation. Initially, two fundamental unit cells with complementary asymmetric stiffness characteristics were devised: a re‐entrant polygonal unit cell possessing “soft in tension, stiff in compression” properties, and a convex polygonal unit cell with “ stiff in tension, soft in compression” behavior. A theoretical model was established to elucidate the mechanism governing self‐contact stiffness modulation, and the model's predictions were subsequently validated by numerical simulation and experimental testing. Furthermore, the potential applications of this metamaterial in the domains of information storage, encryption, and dynamic transmission were explored. For static information storage and encryption, an in‐plane array of the metamaterial unit cells enabled the spatial encoding of stiffness distribution. Concurrently, by adjusting the spacing of the self‐contact blocks, the temporal sequence of stiffness mutation for unit cells within the array can be precisely controlled. The feasibility of this approach for information storage and encryption was experimentally verified. Electromagnetic interference comparison tests showed that the mechanical metamaterial maintained intact information storage under electromagnetic fields (outperforming conventional electronic devices), highlighting its high reliability in extreme environments. In the context of dynamic information transmission, a four‐cell metamaterial was constructed by combining the re‐entrant and convex polygonal unit cells. Leveraging the asymmetric stiffness of this metamaterial, symmetric harmonic excitations were converted into directionally controllable asymmetric vibration responses, thus achieving the directional transport of droplets and mass blocks. The research presented herein establishes a foundational framework for advancing the development of novel information processing technologies based on mechanical media.

## 7 Method

### 7.1 Sample Fabrication

The experimental samples were fabricated using Fused Deposition Modeling (FDM) technology, with the equipment being a Wiiboox Company PRO 300X dual‐nozzle 3D printer (Wiiboox 3D Technology Co., Ltd., Nanjing, China). For the study of static mechanical properties and information storage/encryption, the detailed geometric parameters of the unit cells are shown in Figure S1 and Table S1. The printing material selected was a consumer‐grade shape memory polymer 3D printing filament (SMP4D BASIC, Xingyi Technology Co., Ltd., Shenzhen, China), with a filament diameter of 1.75 mm. For the study of dynamic response and droplet transport, to make the metamaterial softer for effective implementation of stretching and compression vibrations, the geometric parameters of the unit cells were adjusted to 2/3 of the corresponding parameters in Table S1. The printing material used was thermoplastic polyurethane (TPU, hardness 90A, Nosbel New Materials Technology (Suzhou) Co., Ltd.), with a filament diameter of 1.75 mm. All samples were printed with consistent parameters: printing temperature of 215°C, layer thickness of 0.25 mm, infill density of 100%, overall printing speed of 40 mm/s, and a nozzle diameter of 0.4 mm.

It should be noted that although SMP4D BASIC and TPU were used for the static and dynamic experiments, respectively, both belong to the shape memory polymer (SMP) material family. The use of different SMPs was primarily based on the distinct stiffness requirements of different functions. The static information storage and encryption experiments require a clear and distinguishable pressure difference between high‐stiffness and low‐stiffness units under quasi‐static compression to reliably trigger the threshold judgment of the pressure sensors. We therefore selected the higher‐modulus SMP4D BASIC (∼2000 MPa). The dynamic transport experiments, on the other hand, require the structure to generate large‐amplitude tension‐compression deformation under harmonic excitation to validate asymmetric‐vibration‐driven directional transport. We therefore selected the lower‐modulus TPU (∼50 MPa), which allows the structure to produce more pronounced asymmetric vibration responses under the same excitation conditions. In addition, using a high‐modulus material would require a significantly larger excitation force, which exceeds the limited power capacity of our shaker—this was also an important consideration for choosing the low‐modulus TPU in the dynamic experiments. It is worth emphasizing that the successful application of both materials demonstrates the material‐independent versatility of the platform—the self‐contact variable stiffness mechanism is inherently a geometric effect and does not depend on a specific base material.

### 7.2 Tensile/Compressive Experimental Tests

The tensile/compressive experiments (including tension/compression of single cells, tension/compression of the four‐cell metamaterial, and compression of the information storage/encryption in‐plane array structure) were completed using a universal testing machine equipped with a 20 kN load cell (Shenzhen Enpuda Industrial System Co., Ltd.). For tensile experiments, both ends of the sample were fixed to the upper and lower fixtures of the testing machine; for compression experiments, the sample was centered between the upper and lower compression fixtures of the testing machine. All experiments were loaded at a rate of 0.5 mm/min. The computer recorded the displacement and load data, based on which the force‐displacement curves were plotted.

### 7.3 Information Storage and Encryption Experiments

The metamaterial for information storage is a 4 × 4 in‐plane array composed of 16 re‐entrant polygonal unit cells (Figure S9a). The initial state of this in‐plane array structure is that all unit cells are in their original configuration (low stiffness, with self‐contact blocks separated). To store the letter “N”, during information encoding, the units in Column 1, Column 4, and at positions (2,2) and (3,3) were reconfigured to the temporary configuration (high stiffness, self‐contact blocks engaged) (Figure S9b). The temporary configuration automatically recovered to the original configuration when heated to 60°C, thus information can be erased by heating the structure to initialize it. After information erasure, new information can be encoded by reconfiguring specific unit cells; for example, when storing the letter “U”, the units in Column 1, Column 4, and at positions (4,2) and (4,3) were reconfigured to the temporary configuration.

The metamaterial for information encryption also uses a 4 × 4 in‐plane array structure (Figure S10a). To increase design freedom, its basic unit is composed of two re‐entrant polygonal unit cells connected in series (Figure S10b). The initial state of the in‐plane array structure is that all unit cells are in their original configuration (labeled as unit 4), with the preset distances δ₁ and δ_2_ between the self‐contact blocks of each unit cell being equal, both at 1.5 mm. To achieve specific timing actions, specific units in the array need to be geometrically reconfigured (labeled as units 1–3 after reconstruction), with their δ₁ and δ_2_ values shown in Figure S10b. For example, when encrypting the letter “U”, units 1–4 were arranged into a 4 × 4 in‐plane array as shown in Figure S10a. At this point, the in‐plane array metamaterial stores both the letter “U” and ambiguous information “I” and “L”, where the image formed by units 1, 2, and 3 is “U”, the image formed by unit 1 is “I”, and the image formed by units 1 and 2 is “L”.

The information reading system consists of a pressure sensor array, a voltage conversion module, and an LED array (Figure S11a). The 4 × 4 pressure sensor array was made by adhering 16 thin‐film pressure sensors (model RX‐S2822, range 2–50 kg) to the surface of an iron plate using 3 M double‐sided tape. Before adhesion, the surface of the iron plate was wiped with alcohol to remove oil stains and ensure good adhesion. To achieve real‐time information visualization, the pressure sensor array was connected to a 4 × 4 LED matrix via a voltage conversion module. The connection followed a coordinate correspondence rule, meaning the pressure sensor array (row *i*, column *j*) and the LED matrix (row *i*, column *j*) form a one‐to‐one connection. The rated voltage of the LEDs was 5 V. The connection method of the pressure sensors, voltage conversion module, and LEDs is shown in Figure S11b. The pressure sensing threshold (20‐500N) can be adjusted via the voltage conversion module, which has a rated output voltage of 5 V. When the pressure on the sensor is below the set threshold, the module outputs 0 V, and the corresponding LED remains off. When the pressure reaches or exceeds the threshold, the module outputs 5 V, and the corresponding LED is illuminated (as shown in Figure 3b(iv)). The specific operation for visual decoding is as follows: place the information storage/encryption array structure above the pressure sensor array, ensuring each sensor only bears the pressure from the corresponding unit. Then, apply a uniform displacement load *u_y_* to the upper surface of the array structure using the compression fixture of the universal testing machine (Figure S11c). Finally, information is read based on the illumination state of the LED matrix (Figure S11d).

This study adopted differentiated threshold setting strategies for the different requirements of information storage and encryption. For the information storage experiments, the threshold of the voltage conversion module was set with the maximum pressure value of the low‐stiffness units (approx. 120N) as the lower limit and the maximum pressure value of the high‐stiffness units (approx. 500N) as the upper limit; this study set it to 150N (Figure 3b(i)). For the information encryption experiments, combined with the force‐displacement curves of each unit cell (Figure 4b), the pressure threshold *F_T_* of the voltage conversion module was set to 80N and the displacement load *u_y_* to 1.2–1.5 mm as the decryption key. The key feature of this password combination is that only within the 1.2∼1.5 mm displacement interval do the pressures of all target units exceed the threshold; if the displacement deviates from this interval, either too few target units are triggered, or non‐target units are mistakenly triggered, making it impossible to form valid information. Here, 80 N is the correct threshold, while an incorrect threshold of 130N was set to demonstrate the situation where the LED matrix displays ambiguous information when the threshold is wrong.

### 7.4 Vibration Test Experiments for Directional Substance Transport

The equipment used in the vibration test system is shown in Figure S11a, mainly consisting of a YE1311 signal generator, a YE5852B charge amplifier, a JZK series electrodynamic shaker, a SIEMENS data acquisition system, a computer, and a high‐speed camera. The installation of the specimen is shown in Figure S11b. The left end of the directional substance transport metamaterial was fixed to the shaker table via a custom fixture, and the right end of the metamaterial (i.e., the platform end) was suspended by elastic cords to ensure the metamaterial was level, thereby eliminating the influence of bending deformation caused by gravity on the vibration response. During the experiment, the signal generator was set to output an 80 Hz sinusoidal excitation signal. Its output signal was passed through an amplifier to the horizontally placed shaker, thereby applying a horizontal vibration excitation to the specimen. During the experiment, a high‐speed camera was used to record the deformation of the specimen at various moments. Finally, by post‐processing the images, the displacement‐time curves of the input end (left end) and output end (right end) of the metamaterial were obtained. Using a high‐speed camera avoids interference from contact sensors on the metamaterial and allows for obtaining clear vibration deformation diagrams (Figure S12).

### 7.5 Droplet Directional Transport Experiments

To verify the droplet directional transport function, a layer of superhydrophobic nanomaterial (NC‐318, Naroco New Materials Co., Ltd., Changzhou, China) was coated on the surface of the metamaterial platform to reduce droplet adhesion (Figure S16). During the experiment, a droplet was dripped onto the vibrating platform using a dropper, thereby achieving directional actuation of the droplet. To conduct the U‐shaped groove droplet transport experiment, a silicone U‐shaped groove (cross‐sectional width of 6 mm, radius of 3 mm) (Figure 6a) was fixed to the output platform of the metamaterial, causing it to undergo asymmetric vibrations with the platform. A droplet was then dripped into the U‐shaped groove with a dropper to achieve its directional transport. For reverse verification that stiffness asymmetry is key to achieving directional actuation, a control group was set up. The geometric configuration of the control group metamaterial was the same as the temporary configuration, but all its self‐contact blocks were bonded and fixed with epoxy resin adhesive (Figure S17) to lock their relative motion, thus making its stiffness symmetric during tension and compression. Subsequently, under excitation conditions identical to the experimental group (harmonic vibration input at the left end with amplitude 0.6 mm and frequency 80 Hz), a droplet transport experiment was conducted in the U‐shaped groove to observe its transport effect.

### 7.6 Mass Block Directional Transport Experiments

To verify the mass block directional transport function of the metamaterial, the following experimental plan was designed. A thin, long stainless‐steel rod with a diameter of 1.5 mm was fixed to the platform at the output end of the metamaterial to serve as a guide rod for the mass block. The mass block was composed of a silicone sheet with a central hole and an iron ring (Figure S21), with a total mass of approximately 7 g. The inner hole of the silicone sheet provided moderate friction with the stainless‐steel rod, and the static friction force was measured to be approximately 0.6 N. The driving effects in both horizontal and vertical directions were verified through experiments. In the horizontal driving experiment, the system was adjusted to keep the metamaterial and guide rod horizontal. A harmonic excitation was applied to the metamaterial, and the mass block underwent directional motion along the rod under the effect of asymmetric vibrations. In the vertical driving experiment, the metamaterial (temporary configuration) was installed vertically. Under the same excitation conditions, the antigravity upward directional motion of the mass block along the rod was successfully observed, with the excitation acceleration amplitude at the input end measured as approximately 13 G_0_ and the acceleration amplitudes at the output end in the two directions as approximately 32 G_0_ and 61 G_0_ respectively (see Figure S22). The experiment was repeated three times under identical conditions, and the mass block consistently moved upward in all trials, confirming the repeatability of the directional transport.

### 7.7 Numerical Simulation Method

This study conducted a series of finite element simulations using the commercial finite element analysis software COMSOL Multiphysics 6.2, including “Stationary Study” for static analysis and “Time‐Dependent Study” for dynamic response analysis in the Solid Mechanics module. The structure was meshed with free tetrahedron. A mesh convergence study was performed by progressively refining the mesh (Figure S23), comparing the displacement‐time curves obtained with minimum element sizes of 3, 2, 1, 0.5, and 0.25 mm. The results showed that the solutions at 0.5 and 0.25 mm were nearly identical. Considering both computational accuracy and cost, a minimum element size of 0.5 mm was selected for all simulations. The contact between self‐contact blocks was defined as face‐to‐face contact, with the two interacting surfaces of the contact blocks in each unit cell (Figure 1a) set as the source and target surfaces, respectively. The penalty method was employed for normal contact constraint with the penalty factor set to 1 (default value). The friction coefficient was set to zero, since the stiffness switching is dominated by normal contact constraints rather than tangential friction. Furthermore, all analyses considered geometric nonlinearity to accommodate the large bending deformations experienced by the curved beams during loading. An isotropic linear elastic constitutive model was adopted, with material parameters including elastic modulus, density, and Poisson's ratio. For SMP4D, Poisson's ratio was 0.33, density was 1100 kg/m^3^, and Young's modulus was 2000 MPa; for TPU, Poisson's ratio was 0.4, density was 1200 kg/m^3^, and Young's modulus was 50 MPa. For static analysis, one end of the structure was fixed, and a linearly increasing displacement load was applied to the other end. For example, during single‐cell tension, one end was fixed, and a displacement load increasing from 0 to 1 mm was applied to the other end along the axial direction, with a step size of 0.02 mm. For time‐dependent analysis, a displacement excitation with an amplitude of 0.6 mm and a frequency of 80 Hz was applied to the left end of the metamaterial. The solver type was set to implicit, the time integration method was Generalized alpha, the step size was set to free, and the calculation time was 0.15s. In addition, to comprehensively characterize the dynamic behavior of the structure, frequency sweep (20∼300 Hz) and damping sensitivity analyses were also performed on the temporary configuration of the metamaterial for directional transport. The detailed settings are provided in Section S5.

## Author Contributions

**Huadong Qin**: Writing – original draft, Validation, Investigation, Data curation. **Wenyu Zhu**: Investigation, Visualization, Methodology. **Xin Wang**: Conceptualization, Methodology, Writing – review & editing. **Cheng Shen**: Writing – review & editing, Data curation, Supervision. **Li Cheng**: Methodology, Supervision, Writing – review & editing, Tianjian Lu: Writing – review & editing, Investigation, Supervision. **Terence Xiaoteng Liu**: Investigation, Data curation, Writing – review & editing. **Han Meng**: Writing – review & editing, Writing – original draft, Validation, Methodology, Investigation, Formal analysis, Data curation, Supervision, Project administration.

## Conflicts of Interest

The authors declare that they have no conflicts of interest.

## Supporting information




**Supporting File 1**: advs77680‐sup‐0001‐SuppMat.docx.

**Supporting File 2**: advs77680‐sup‐0002‐MovieS1.mp4.

**Supporting File 3**: advs77680‐sup‐0003‐MovieS2.mp4.

**Supporting File 4**: advs77680‐sup‐0004‐MovieS3.mp4.

**Supporting File 5**: advs77680‐sup‐0005‐MovieS4.mp4.

**Supporting File 6**: advs77680‐sup‐0006‐MovieS5.mp4.

**Supporting File 7**: advs77680‐sup‐0007‐MovieS6.mp4.

**Supporting File 8**: advs77680‐sup‐0008‐MovieS7.mp4.

**Supporting File 9**: advs77680‐sup‐0009‐MovieS8.mp4.

**Supporting File 10**: advs77680‐sup‐0010‐MovieS9.mp4.

## Data Availability

The data that support the findings of this study are available from the corresponding author upon reasonable request.
