# Supplementary material for: Programmable Information Processing With Reconfigurable Self‐Contact Variable Stiffness Mechanical Metamaterial
Source: Adv Sci (Weinh). 2026 Sep 8:e77680. Online ahead of print. doi: 10.1002/advs.77680 (PMC13554505; doi:10.1002/advs.77680)
Supplement: Supplementary file 1 [file ADVS-9999-e77680-s009.docx]

**Supporting Information**

**Programmable Information Processing with Reconfigurable Self-Contact Variable Stiffness Mechanical** **Metamaterial**

Huadong Qin^1,2^, Wenyu Zhu^1,2^, Xin Wang^3^, Cheng Shen^1,2^, Li Cheng^4^, Tian Jian Lu^1,2^, Terence Xiaoteng Liu^5^ and Han Meng^1,2^^[1]^

*^1^State Key Laboratory of Mechanics and Control for Aerospace Structures, Nanjing University of Aeronautics and Astronautics, Nanjing 210016, P.R. China*

*^2^MIIT Key Laboratory of Multifunctional Lightweight Materials and Structures, Nanjing University of Aeronautics and Astronautics, Nanjing, 210016, P.R. China*

*^3^China Academy of Aerospace System and Innovation, Beijing, 100088 P. R. China*

*^4^Department of Mechanical Engineering, The Hong Kong Polytechnic University, Hong Kong, China*

*^5^School of Engineering, Physics and Mathematics, Northumbria University, Newcastle upon Tyne, U.K. NE1 8ST*

**Files:**

Section S1. Geometric design of unit cell

Section S2. Boundary conditions and internal force equations

Section S3. Unit cell static mechanical properties

Section S4. Theoretical storage density at the manufacturing limit

Section S5. **Frequency sweep and damping sensitivity analysis**

**Supplementary Figures:**

Fig.S9 to S23

**Movies:**

Movie S1. Heating and shape recovery of re-entrant unit cell

Movie S2. Visualization of stored information

Movie S3. Information encryption

Movie S4. Effect of electromagnetic interference on an electronic storage device

Movie S5. Effect of electromagnetic interference on the information storage metastructure

Movie S6. Directional droplet transport in the U-shaped groove

Movie S7. Control experiment on directional droplet transport

Movie S8. Mass block transport in horizontal and vertical directions

Movie S9. Directional droplet transport on the platform

**Section S1. Geometric design of unit cell**

Fig. S1 Geometric design. (a-c) Parameter diagrams for re-entrant and convex unit cells, and photographs of samples.

Table S1 Geometric Parameters

| Parameter | Value (mm) |
| --- | --- |
| $h_{1}$ | 21 |
| $h_{2}$ | 30 |
| $h_{3}$/$h_{3}^{'}$ | 6/6.75 |
| $h_{4}$/$h_{4}^{'}$ | 9/7.5 |
| $b_{1}$/$b_{1}^{'}$ | 10.5/16.5 |
| $b_{2}$/$b_{2}^{'}$ | 13.5/3 |
| $b_{3}$/$b_{3}^{'}$ | 22.5/7.5 |
| $b_{4}$/$b_{4}^{'}$ | 31.5/19.5 |
| $\delta$ | 1.5 |
| $\theta$ | 45° |
| $t$ | 1.5 |

**Section S2.** **Boundary conditions and internal force equations**

Fig.S2 Force diagrams of the inclined beam. (a) Force diagram of the inclined beam when the unit cell is under compression. (b) Force diagram of the inclined beam when the unit cell is under tension. (c) Various unit loads required for calculating deformation.

**Internal Force Equations for the Calculation Process**

Bending moment equation of the inclined beam under the unit load $\bar{M}$ is

$\overline{M_{1}}(x)=-1$ (S1)

Bending moment equation of the inclined beam under the load $F_{c}$ is

$M_{1}(x)=\frac{\sqrt{2}}{2}Fx$ (S2)

Axial force and bending moment equations of the beam under the unit load $\bar{F}_{c}$ are given as

$\overline{F}_{N}=-\frac{\sqrt{2}}{2}$ (S3)

$\overline{M}(x)=\frac{\sqrt{2}}{2}x$ (S4)

Axial force and bending moment equations of the beam under the actual loads $F_{c}$ and $X_{1}^{C}$ are

$F_{N}=-\frac{\sqrt{2}}{2}F$ (S5)

$M(x)=\frac{\sqrt{2}}{2}Fx-X_{1}^{C}$ (S6)

Axial force and bending moment equations of the inclined beam under the unit load $\bar{F}_{x}$ are

$\overline{F}_{N2}=\frac{\sqrt{2}}{2}$ (S7)

$\overline{M_{2}}(x)=\frac{\sqrt{2}}{2}x$ (S8)

Bending moment equation of the inclined beam under the unit load $\bar{M}$ is given as

$\overline{M_{3}}(x)=1$ (S9)

Axial force and bending moment equations of the inclined beam under the load $F_{t}$ are

$F_{N2}=\frac{\sqrt{2}}{2}F$ (S10)

$M_{2}(x)=-\frac{\sqrt{2}}{2}Fx$ (S11)

Axial force and bending moment equations of the beam under the unit load $\bar{F}_{t}$ are

$\overline{F}_{N}^{t}=\frac{\sqrt{2}}{2}$ (S12)

$\overline{M^{t}}(x)=-\frac{\sqrt{2}}{2}x$ (S13)

Axial force and bending moment equations of the beam under the actual loads $F_{t}$, $X_{1}$ and $X_{2}$ are

$F_{N}^{t}=\frac{\sqrt{2}}{2}(F+X_{2})$ (S14)

$M^{t}(x)=\frac{\sqrt{2}}{2}(X_{2}-F)x+X_{1}$ (S15)

**Section S3.** **Unit cell static mechanical properties**

Fig.S3 Deformation modes of the convex unit cell under 1 mm tensile/compressive loads. (a, d) Compression. (b, e) Undeformed state. (c, f) Tension. The top row (a-c) shows the Original configuration, while the bottom row (d-f) shows the Temporary configuration.

Fig.S3 shows the deformation diagrams of the Convex Unit Cell under 1 mm tension or compression. Due to its positive Poisson’s ratio, the structure undergoes lateral contraction under tensile loading, which further compresses the pre‑closed contact blocks in the temporary configuration and establishes effective internal constraints (Fig.S3f). This is the fundamental reason for its significantly increased tensile stiffness (594.81 N/mm). In contrast, under compressive loading, lateral expansion of the structure causes the contact blocks to separate (Fig.S3d), and thus no effective internal constraint is established. As a result, its compressive stiffness (130.65 N/mm) remains at a level similar to that of the original configuration. This switching of the constraint mechanism based on contact state under tension/compression clearly reveals the intrinsic mechanism behind the “stiff in tension, soft in compression” behavior of the temporary configuration. In the original configuration, the contact blocks (with a preset gap $\delta=1.5$ mm) remain open during the limited deformation (1 mm), so its tensile and compressive stiffnesses are comparable (Fig.S3a, c).

Fig.S4 Force-deformation response of the re-entrant unit cell.

Fig.S5 Deformation modes of the re-entrant unit cell. (a, d) Under 1 mm compressive load. (b, e) Undeformed state. (c, f) Under 1 mm tensile load. The top (a-c) and bottom (d-f) rows correspond to the Original and Temporary configurations, respectively.

Fig.S4 shows the force–deformation curves of the Re-entrant Unit Cell under tension and compression for both its configurations. As can be seen from the figure, the compressive stiffness of the temporary configuration (purple curve) is significantly greater than that of the other loading cases. The stiffness of the structure can be obtained by calculating the slope of the force–deformation curve. The compressive stiffness $k_{c}^{R}$ of the Re-entrant Unit Cell in its temporary configuration is 532.18 N/mm, whereas the stiffness of the other three cases (tension and compression of the original configuration, and tension of the temporary configuration) are very close, ranging between 104.95 and 121.42 N/mm. The former is about five times the latter, indicating a pronounced stiffness‑switching effect. Moreover, the tensile stiffness $k_{t}^{R}$ of the temporary configuration is 121.42 N/mm, which is much lower than its compressive stiffness $k_{c}^{R}$ exhibiting an asymmetric “soft in tension, stiff in compression” characteristic (the deformation patterns are shown in Fig.S5). This behavior complements the “stiff in tension, soft in compression” characteristic of the Convex Unit Cell.

**Section S4.** **Theoretical storage density at the manufacturing limit**

As illustrated in Fig. S6, we set the beam thickness $t^{'}$ equal to the minimum feature size 0.1 mm by 3D printing, and follow these design constraints to ensure mechanical functionality: The inclined beam must behave as a slender member with a length-to-thickness ratio of at least 5, which yields a minimum beam length of $5t^{'}=0.5 \mathrm{mm}$; The contact blocks must be considerably stiffer than the inclined beams to avoid significant deformation upon contact, the minimum contact-block block thickness is therefore set to $3t^{'}=0.3 \mathrm{mm}$; The inclination angle of the inclined beam is $\theta=45^{\circ}$.

Based on these constraints, the minimum achievable unit-cell width is obtained as:

(S16)

and the minimum unit-cell thickness is $a=0.1 \mathrm{mm}$. Substituting these values into Eq. (14), the theoretical storage density is approximately 465.9 KB/m².

Fig.S6 Schematic illustration of the metamaterial unit cell.

**Section S5. Frequency sweep and damping sensitivity analysis**

To comprehensively characterize the dynamic behavior of the proposed metamaterial, we performed a frequency sweep on the temporary configuration of the metamaterial for directional transport over the range of 20~300 Hz. At each frequency, a transient analysis was conducted with a constant displacement amplitude applied at the input end. The positive and negative displacement amplitudes at the output end were extracted and plotted as functions of excitation frequency (Fig. S7f). Additionally, the displacement-time curves at five representative frequencies (30 Hz, 40 Hz, 86 Hz, 100 Hz, and 300 Hz) are presented (Fig. S7a–e).

**Fig.S7** Frequency sweep analysis of the temporary configuration. (a–e) Displacement-time curves at representative frequencies: (a) 30 Hz, (b) 40 Hz, (c) 86 Hz, (d) 100 Hz, and (e) 300 Hz. (f) Positive and negative displacement amplitudes at the output end as functions of excitation frequency (20–300 Hz, step size 10 Hz).

The sweep results show that both the positive and negative displacement amplitudes first increase and then decrease as the excitation frequency increases, reaching a peak at approximately 86 Hz, which corresponds to the resonance response of the structure (Fig. S7f). At the resonance frequency, the difference between the positive and negative amplitudes is most pronounced (Fig. S7c). It is worth noting that the asymmetric vibration is not confined to the resonance region: away from resonance, although the vibration amplitude decreases, a difference between the positive and negative displacement amplitudes still persists

Rayleigh damping was employed in the transient analyses, with the damping matrix expressed as $\mathbf{C} = \alpha\mathbf{M} + \beta\mathbf{K}$, where $\mathbf{M}$ is the mass matrix and $\mathbf{K}$ is the stiffness matrix. To evaluate the sensitivity of the vibration response to damping, we compared the responses under four different Rayleigh damping coefficients: (i) $\alpha=10$ s⁻¹, $\beta=1\times10$⁻⁵ s, (ii) $\alpha=40$ s⁻¹, $\beta=4\times10⁻⁵$ s, (iii) $\alpha=70$ s⁻¹, $\beta=7\times10⁻⁵$ s, and (iv) $\alpha=100$ s⁻¹, $\beta=1\times10⁻4$ s (Fig. S8). The results show that as the damping coefficient increases, the response amplitude decreases, while the overall trend and asymmetric characteristics of the vibration remain unchanged. This confirms that the directionality of the asymmetric response is governed by the tension-compression stiffness asymmetry of the structure, rather than by damping.

Fig.S8 Damping sensitivity analysis. Comparison of output displacement responses under different Rayleigh damping coefficients: (i) $\alpha=10$ s⁻¹, $\beta=1\times10$⁻⁵ s, (ii) $\alpha=40$ s⁻¹, $\beta=4\times10⁻⁵$ s, (iii) $\alpha=70$ s⁻¹, $\beta=7\times10⁻⁵$ s, and (iv) $\alpha=100$ s⁻¹, $\beta=1\times10⁻4$ s.

**Supplementary Figures**

Fig.S9 In-plane array structure. (a) Information storage array. (b) Open and closed states of each unit in the array when encoding the letter "N".

Fig.S10 In-plane array structure. (a) Information encryption array. (b) Four basic units in the array.

Fig.S11 Information Visualization System. (a) Components of the system. (b) Circuit schematic of pressure sensor, voltage conversion module and LED. (c) Experimental setup for information decoding. (d) Encoding and decoding results.

Fig.S12 Information encryption array. (a) In-plane array structure after selective reconstruction. (b) Pressure on the substrate, showing that an incorrect threshold setting results in the output of invalid information "L".

Fig.S13 Deformation Images: Experiment vs. FEA, (a) original configuration, (b) temporary configuration.

Fig.S14 Vibration Experiment. (a) Vibration Testing System. (b) Installation method of the sample.

Fig.S15 Deformation images captured by high-speed camera. Maximum Tensile and Compressive Deformation of (a) the original configuration and (b)the temporary configuration.

Fig.S16 Schematic of the directional droplet transport platform. (a) original configuration, (b) temporary configuration. The directional manipulation of droplets can be achieved directly on the surface of the metastructure platform (see details in Fig.S18-S19 and Movie S9, Supporting Information) without relying on an external channel.

Fig.S17 Sample of control group.

Fig.S18 Directional droplet transport experiment on the platform of the Directional Droplet Transport Metamaterial (DDTM) in its original configuration (droplets moving right).

Fig.S19 Directional droplet transport experiment on the platform of the DDTM’s temporary configuration (droplets moving left)

Fig.S20 Schematic diagram of droplet sorting.

Fig.S21 Stick-slip drive experimental sample.

**Fig. S22 Acceleration response curves at the input and output ends in the anti-gravity transport experiment.**

Fig.S23 Mesh convergence study. Comparison of displacement-time curves obtained with different minimum element sizes (3 mm, 2 mm, 1 mm, 0.5 mm, and 0.25 mm).

1. Corresponding author.

   *Email address*: [menghan@nuaa.edu.cn](mailto:menghan@nuaa.edu.cn) (Han Meng)
